# Transcriptomic and genomic studies classify NKL54 as a histone deacetylase inhibitor with indirect influence on MEF2-dependent transcription

**DOI:** 10.1093/nar/gkac081

**Published:** 2022-02-12

**Authors:** Martina Minisini, Eros Di Giorgio, Emanuela Kerschbamer, Emiliano Dalla, Massimo Faggiani, Elisa Franforte, Franz-Josef Meyer-Almes, Rino Ragno, Lorenzo Antonini, Antonello Mai, Francesco Fiorentino, Dante Rotili, Monica Chinellato, Stefano Perin, Laura Cendron, Christian X Weichenberger, Alessandro Angelini, Claudio Brancolini

**Affiliations:** Department of Medicine, Università degli Studi di Udine. P.le Kolbe 4, 33100 Udine Italy; Department of Medicine, Università degli Studi di Udine. P.le Kolbe 4, 33100 Udine Italy; Institute for Biomedicine, Eurac Research, Affiliated Institute of the University of Lübeck. Via Galvani 31, 39100 Bolzano, Italy; Department of Medicine, Università degli Studi di Udine. P.le Kolbe 4, 33100 Udine Italy; Department of Medicine, Università degli Studi di Udine. P.le Kolbe 4, 33100 Udine Italy; Department of Medicine, Università degli Studi di Udine. P.le Kolbe 4, 33100 Udine Italy; Department of Chemical Engineering and Biotechnology, University of Applied Science, Haardtring 100, 64295 Darmstadt, Germany; Rome Center for Molecular Design, Department of Chemistry and Technology of Drugs, “Sapienza” University of Rome, Piazzale Aldo Moro 5, Rome 00185, Italy; Rome Center for Molecular Design, Department of Chemistry and Technology of Drugs, “Sapienza” University of Rome, Piazzale Aldo Moro 5, Rome 00185, Italy; Department of Chemistry and Technology of Drugs, “Sapienza” University of Rome, Piazzale Aldo Moro 5, Rome 00185, Italy; Department of Chemistry and Technology of Drugs, “Sapienza” University of Rome, Piazzale Aldo Moro 5, Rome 00185, Italy; Department of Chemistry and Technology of Drugs, “Sapienza” University of Rome, Piazzale Aldo Moro 5, Rome 00185, Italy; Department of Biology, University of Padova, Via U. Bassi, 58/B, 35121 Padova, Italy; Department of Molecular Sciences and Nanosystems, Ca' Foscari University of Venice, Via Torino 155, 30172 Mestre, Italy; European Centre for Living Technology (ECLT), Dorsoduro 3911, Calle Crosera, 30123 Venice, Italy; Department of Biology, University of Padova, Via U. Bassi, 58/B, 35121 Padova, Italy; Institute for Biomedicine, Eurac Research, Affiliated Institute of the University of Lübeck. Via Galvani 31, 39100 Bolzano, Italy; Department of Molecular Sciences and Nanosystems, Ca' Foscari University of Venice, Via Torino 155, 30172 Mestre, Italy; European Centre for Living Technology (ECLT), Dorsoduro 3911, Calle Crosera, 30123 Venice, Italy; Department of Medicine, Università degli Studi di Udine. P.le Kolbe 4, 33100 Udine Italy

## Abstract

In leiomyosarcoma class IIa HDACs (histone deacetylases) bind MEF2 and convert these transcription factors into repressors to sustain proliferation. Disruption of this complex with small molecules should antagonize cancer growth. NKL54, a PAOA (pimeloylanilide o-aminoanilide) derivative, binds a hydrophobic groove of MEF2, which is used as a docking site by class IIa HDACs. However, NKL54 could also act as HDAC inhibitor (HDACI). Therefore, it is unclear which activity is predominant. Here, we show that NKL54 and similar derivatives are unable to release MEF2 from binding to class IIa HDACs. Comparative transcriptomic analysis classifies these molecules as HDACIs strongly related to SAHA/vorinostat. Low expressed genes are upregulated by HDACIs, while abundant genes are repressed. This transcriptional resetting correlates with a reorganization of H3K27 acetylation around the transcription start site (TSS). Among the upregulated genes there are several BH3-only family members, thus explaining the induction of apoptosis. Moreover, NKL54 triggers the upregulation of MEF2 and the downregulation of class IIa HDACs. NKL54 also increases the binding of MEF2D to promoters of genes that are upregulated after treatment. In summary, although NKL54 cannot outcompete MEF2 from binding to class IIa HDACs, it supports MEF2-dependent transcription through several actions, including potentiation of chromatin binding.

## INTRODUCTION

The MEF2 family of transcription factors (TFs) includes four paralogues MEF2A, B, C and D that regulate differentiation and important adaptive responses. They coordinate the expression of a rather large number of genes in a context- and partner-dependent manner (1). Dysregulations of these TFs have been documented in various diseases (1–5). The involvement of MEF2s in various pathological contexts makes them attractive candidates for novel therapeutic approaches aimed at restarting a dysregulated transcriptional program. MEF2 proteins are characterized by the presence of the highly conserved MADS and MEF2 domains in the N-terminal region. These domains are essential for DNA binding, dimerization and interaction with other partners. In contrast, the C-terminal region is much less conserved and is involved in transcriptional activation (1). A hydrophobic groove within the MADS/MEF2 domain contains the binding site for amphipathic α-helices present in some MEF2 partners. Transcriptional repressors, such as class IIa HDACs or Cabin1, as well as activators, for example the histone acetyltransferase p300, bind MEF2 via this mechanism (6,7). A β-sheet organizes the floor of this deep hydrophobic groove, while two helices form the rim (6). In HDAC9, the hydrophobic side of the amphipathic α-helix consisting of Val143, Leu147, Phe150 and Leu151 fits precisely into the hydrophobic groove of MEF2B (8). Similarly, the co-repressor Cabin1 adopts an amphipathic α-helix to bind this hydrophobic groove, forming a triple-helical interaction (6).

The possibility of affecting the transcriptional activity of MEF2 by small molecules that can bind this hydrophobic groove has been exploited in the past (9). A virtual screen identified a series of small molecules belonging to the class of PAOA (pimeloylanilide-o aminoanilides). Starting from the original compound BML-210 ((*N*-(2-aminophenyl)-*N*′ phenyloctanol diamine), which was initially identified as a pan-HDACs inhibitor (10), several analogous compounds with improved solubility were characterized (11).

The MEF2–HDAC axis is frequently circuited in leiomyosarcoma (LMS) a rare group of soft tissue sarcomas (STS), highly aggressive and with few therapeutic options (11–14). In this manuscript, we have investigated the possibility to block LMS proliferation, by targeting the interaction of MEF2 with class IIa HDACs, using PAOA derivatives. We found that PAOA derivatives are potent inhibitors of LMS cell proliferation, however, they are unable to disrupt the binding between MEF2 and class IIa HDACs. Conversely, PAOA derivatives appear to act mainly as inhibitors of zinc-dependent class I HDACs.

## MATERIALS AND METHODS

### Antibodies and chemicals

The primary antibodies used were anti: MEF2D (BD Bioscience); MEF2A (C-21), (Santa Cruz Biotechnology); MEF2C (15); Actin (Sigma-Aldrich); HDAC4 (16); HDAC5 (17); HDAC7 (18); HDAC9 (19); H3K27ac (ab4729) and H3K9ac (ab4441) (Abcam); Histone H3 (H0164, Sigma-Aldrich) HDAC3 (PA5-29026, Invitrogen). The following chemicals were used: SAHA (Cayman Chemicals); TMP195 (MedChemExpress), BML-210 (Sigma-Aldrich). The PAOA derivatives MC2983, MC2984, MC2985 and MC2991 were synthetized. All new compounds had spectral (^1^H NMR, ESI-MS) data in agreement with the structure. Full details of the syntheses will be reported elsewhere. NKL54 (*N*-(2-aminophenyl)-*N*'-[3-(trifluoromethyl)phenyl]heptanediamide) was synthesized by SIA Chemspace (Riga, Latvia).

### Cell cultures and cytofluorimetric analysis

Leiomyosarcomas cells (LMS), SK-UT-1, SK-LMS-1 and DMR were grown as previously described (19). For PI staining, cells were collected and resuspended in 0.1 ml of 10 μg/ml propidium iodide (PI) (Sigma-Aldrich), in PBS and incubated for 10 min at RT. After washes, cells were fixed with 1% formaldehyde (Sigma-Aldrich) and treated with 10 μg/ml RNase A. Fluorescence was determined with a FACScan™ (Beckman Dickinson) and with Countess II FL automated cell counter (Invitrogen).

### Immunoblotting

Cell lysates, after SDS-PAGE and immunoblotting on nitrocellulose (Whatman), were incubated with primary antibodies. HPR-conjugated secondary antibodies were obtained from Sigma-Aldrich and blots were developed with Super Signal West Dura (Thermo Scientific). For antibodies stripping, blots were incubated for 10min with Restore PLUS Western Blot Stripping Buffer (Thermo Scientific).

### Caspase and resazurin reduction assays

The caspase activity was evaluated using the Apo-ONE caspase-3/7 homogeneous assay (Promega). Cells grown in 96-well plates were treated with the different insults and tested for caspase activity as recommended by the vendor. Resazurin assay was done as already described (20). Briefly, cells were incubated for 150 min. at 37°C with resazurin solution (0.15 mg/ml) (Sigma-Aldrich). The product of reduction was quantified by using the PerkinElmer EnSpire 2300 Multilabel Reader.

### Molecular modelling

Three-dimensional atomic coordinates of crystallized MEF2A in complex with DNA and BML-210 were retrieved from the protein data bank using accession code 3MU6. The structure was subjected to a cleaning procedure eliminating all water and non-protein atoms/molecules. During the complex cleaning the hydrogen positions were optimized by means of a single point minimization using the default settings available in UCSF Chimera (version 1.14) (21) using the AmberF14SB force field (22). The cleaned minimized complex was then separated into lock (protein + DNA) and key (BML-210) for the subsequent docking assessment procedure. Smina and Plants programs (23,24) implemented in the Py-Docking web app of 3d-qsar.com portal (2019) were assessed for docking suitability. Experimental conformation re-docking (ECRD) and random conformation re-docking (RCRD) procedures (25–27) indicated the Plants/PLP combination as the best performing. For all docking the program default setting were maintained with an extended docking space of 4 Å (extended grid for Smina and extended radius for Plants). Only the lowest energy conformation was considered for the RMSD evaluation. In the case of Smina the docked conformations were also re-scored by the internal minimization available features.

### HDAC assay

Lysine deacetylase assay was carry-out using the HDAC-Glo I/II assay kit (Promega), following manufacturer specifications. Briefly, native lysates were generated from 1.0 × 10^5^ SK-UT-1 cells, previously incubated for 4 h with HDAC inhibitors. The luminescence was quantified by using the Modulus II microplate multimode reader (Turner Biosystem).

For the *in vitro* enzyme activity assays, recombinant HDAC4 and 8 were produced as described previously (28). The other HDAC isoenzymes were purchased from BPS Bioscience. To determine the inhibitory effect of compounds on HDACs, 1 nM of the respective HDAC isozyme was incubated with a serial dilution of the compounds for 30 min at 30°C in the assay buffer (25 mM Tris–HCl, pH 8.0, 75 mM KCl, 0.001% v/v Pluronic F-127). The catalytic reaction was carried out by the addition of 50 μM of the substrate Boc-Lys(Ac)-AMC for HDACs 1, 2, 3, and 6 or 20 μM of the substrate Boc-Lys(trifluoracetyl)-AMC for HDACs 4 and 8 followed by an incubation for 60 min at 30°C. The reaction was stopped by adding 40 μM SAHA for HDACs 1, 2, 3 and 6 or 20 μM SATFMK for HDACs 4 and 8. The deacetylated substrate was converted into the fluorescent product AMC by the addition of 0.5 mg/ml trypsin. The release of the AMC was followed in a microplate reader (excitation: 360 nm, emission: 460 nm; PHERAstar FS, BMG LABTECH) and then correlated to enzyme activity. All obtained dose–response curves were fitted to a four parameter fit model provided by Prism 6 yielding the IC_50_-value

### Protein expression and purification

MEF2A (1–92) and MEF2D (1–95) were cloned into pRham and pETite vectors (Lucigen), respectively. Both proteins were expressed using *Escherichia coli* T7 SHuffle cells (NEB) and growth in Terrific Broth (TB) media. Expression of MEF2A and MEF2D was induced at OD_600_ = 0.8 by adding 0.2% (w/v) rhamnose and 1 mM isopropyl-β-d-1thiogalactopyranoside (IPTG), respectively. Induced cells were maintained at 28°C overnight. Cell pellets were resuspended in lysis buffer (10 mM HEPES pH 7.7, 30 mM NaCl, 0.5 mM EDTA, 0.5 mM DTT) and processed by French press. Both MEF2A and MEF2D proteins were purified via ion exchange chromatography using 20 ml of SP-Sepharose resin (GE Healthcare) equilibrated with 10 mM HEPES, pH 7.7. Elution was achieved by applying a 0–2 M (NH_4_)_2_SO_4_ linear gradient. Eluted fractions were collected and further purified using a HiPrep Butyl FF 16/10 column (GE Healthcare) equilibrated with 10 mM HEPES pH 7.7, 2 M (NH_4_)_2_SO_4_, 0.5 mM EDTA, 0.5 mM DTT. Elution was performed by applying a 2–0 M salt gradient. Purest fractions were collected and loaded on a HiLoad Superdex 75 16/60 column (GE Healthcare), equilibrated with storage buffer 10 mM HEPES pH 7.7, 200 mM NaCl, 0.5 mM EDTA, 0.5 mM DTT, 10% v/v glycerol). Both proteins were concentrated to 66 μM, aliquoted and stored at −80°C.

### Chemical synthesis of peptides

Peptides pHDAC4 (aa 170–183; AcNH-GSGEVKMKLQEFVLNKK-CONH_2_) and F-pHDAC4 (aa 170–183, fluorescein-GSGEVKMKLQEFVLNKK-CONH_2_) were synthesized by standard Fmoc (9-fluorenylmethoxycarbonyl) solid-phase peptide synthesis (SPPS). Fmoc-protected amino acids, PyBOP, 5(6)-carboxyfluorescein, acetic anhydride, anisole, dichloromethane (DCM) and *N,N*-dimethylformamide (DMF) and Rink Amide MBHA resin (100–200 mesh, loading 0.4–0.9 mmol g^−1^ resin, 0.01 mmol scale) were purchased from Novabiochem. Acetonitrile (ACN), formic acid, *N*-methylmorpholine (NMM), octanedithiol (ODT), piperidine, trifluoroacetic acid (TFA) and thioanisole were purchased from Sigma-Aldrich. *N*-methylpirrolidone (NMP) was purchased from VWR. All chemicals were used as received without further purification. Peptides were prepared using a MultiPep RSi peptide synthesiser (Intavis). Fmoc groups were removed using a 20% v/v solution of piperidine in DMF (180 μl × 2). Amino acid coupling was carried out twice for each Fmoc-amino acid (7.5 eq., 0.5 M solution in DMF). Final acetylation capping was performed using a 5% (v/v) solution of acetic anhydride in DMF. DCM washes (0.3 ml × 5) were performed at the end of synthetic process. NMP was used as cosolvent in the peptide synthesis. A 4 M NMM solution in DMF was added as weak base for Fmoc deprotection. The final peptides were deprotected (side-chain protected groups) and cleaved from the resin using a TFA/thioanisole/H_2_O/anisole/ODT mixture (90/2.5/2.5/2.5/2.5% v/v) for 3 h at room temperature. The resin was removed by filtration under vacuum and the peptides were precipitated with cold diethyl ether (50 ml). The precipitated peptides were resuspended in diethyl ether (30 ml × 2) and centrifuged (3 times). Finally, the peptides were dissolved in H_2_O:ACN (1:1), freeze-dried and lyophilized. Crude peptides were dissolved in DMSO and purified by preparative reversed-phase high performance liquid chromatography (RP-HPLC) on a Waters Delta Prep LC 4000 System equipped with Waters 2489 dual λ absorbance detector and with both Waters 600 pump, PrepLC Controller (Waters) and a C18 SymmetryPrep (Waters) functionalized silica column (7 μm, 19 mm × 150 mm). At a flow rate of 20 ml min^−1^, a linear gradient (10% to 50% in 35 min) was applied with a mobile phase composed of eluant A (99.9% v/v H_2_O, 0.1% v/v TFA) and eluant B (99.9% v/v ACN and 0.1% v/v TFA). The purified peptides were freeze-dried. The purity and molecular mass of the peptides was assessed by LC-ESI as described below. Concentrations of peptides were determined by UV spectrophotometry.

### Mass spectrometric analysis

The molecular mass of each peptide was determined by electrospray ionisation mass spectrometry (ESI–MS) performed on a single quadrupole liquid chromatograph InfinityLab LC/MSD mass spectrometer (InfinityLab LC/MSD, Agilent) coupled to a 1260 Infinity II LC system, Agilent). The reversed-phase HPLC column was a Nucleosil 100-5 C18 Macherey-Nagel (5 μm, 125 mm × 4 mm). The system operated with the standard ESI source and in the positive ionisation mode. For mass spectrometric analysis, the samples were mixed with 50% (v/v) ACN, 50% (v/v) H_2_O. Peptides were run at a flow rate of 1 ml min^−1^ with a linear gradient of solvent B over 15 min (A: 99.9% v/v H_2_O and 0.1% v/v formic acid; B: 99.9% v/v ACN and 0.1% v/v formic acid). Data were acquired, processed and analysed using Agilent OpenLAB CDS (Agilent Technologies) and MestReNova (Mestrelab Research S.L.) software.

### Fluorescence polarization binding assay

Fluorescence polarization (*FP*) values were determined using the Equation (1), where *S* is the fluorescence intensity of emitted light parallel to excitation, *P* is the fluorescence intensity of emitted light perpendicular to excitation, and *G* is the correction factor that correct for instrument bias.

(1) $$\begin{equation*}FP = 1000\end{equation*}$$

The *G* factor was experimentally determined using the probe alone.

### Fluorescence polarization assays, direct binding

Proteins were diluted in 10 mM HEPES, 200 mM NaCl, 1 mM DTT, 1 mM EDTA, pH 7.7 at final concentrations ranging from 0.5 to 300 μM. Titration assays were performed using fluorescently labelled peptide F-pHDAC4 at a final concentration of 0.26 μM. Each mixture (100μl) was transferred into black 96-well microplates (Optiplate, PerkinElmer) and incubated at room temperature for 1 h. Polarization signals were recorded at 25°C using an EnVision Multlabel Plate Reader (PerkinElmer) with an excitation filter at 480 nm, an emission filter at 535 nm and a 505 nm dichroic mirror. Orbital shaking (200 rpm for 0.1 s) was applied. The average fluorescence polarization values of at least three independent experiments were plotted as a function of MEF2A or MEF2D concentration. Equilibrium dissociation constants (*K_D_*) were determined by non-linear regression analyses of polarization (*FP*) versus the total protein concentration (*P*_T_) using Equation (2):

(2) $$\begin{eqnarray*}F\ &=& {F_L}\ + \left( {\frac{{{F_{LP}} - {F_L}}}{{2{L_T}}}} \right)({L_T} + {P_T} + {K_D} \nonumber\\ &&- \sqrt {{{\left( {{L_T} + {P_T} + {K_D}} \right)}^2} - 4{L_T}{P_T}} \end{eqnarray*}$$

where *F* is the measured average fluorescence polarization, *F*_L_ is the fluorescence polarization of free labelled peptide, *F*_LP_ is the maximum fluorescence polarization of the peptide–protein complex and *L*_T_ represents the total labelled peptide concentration.

### Fluorescence polarization assays, competition

Protein binding by different compounds was measured by incubating different concentrations of each ligand (5-fold dilutions, ranging from 10 to 250 μM) with 0.26 μM F-pHDAC4 and 20 μM of MEF2A or MEF2D. Unlabelled pHDAC4 peptide (3-fold dilutions ranging from 1 to 100 μM) was used as positive control. Each mixture (100 μl) containing 10 mM HEPES, 200 mM NaCl, 1 mM DTT, 1 mM EDTA, pH 7.7, 0.26 μM F-pHDAC4, 20 μM of protein and the ligand of interest was transferred into black 96-well microplates (Optiplate, PerkinElmer) and incubated at room temperature for 1 and 24 h. Controls samples without proteins and without ligands were also prepared to estimate fluorescence of displaced and bound probe, respectively. Polarization signals were recorded at 25°C using an EnVision Multlabel Plate Reader (PerkinElmer) as described above. The average fluorescence polarization values of at least three independent experiments were plotted as a function of ligand concentration. Equation (3) was applied to determine the half-maximal inhibitory concentration (*IC*_50_) of each molecule:

(3) $$\begin{equation*}F\ = {F_L}\ + \frac{{\left( {{F_{LP}} - {F_L}} \right)}}{{1 + \frac{I}{{IC50}}}}\end{equation*}$$

where *F* is the measured average fluorescence polarization, *F*_L_ is the fluorescence polarization of free labelled peptide, *F*_LP_ is the maximum fluorescence polarization of the peptide–protein complex and *I* is the concentration of the compound. Finally, the inhibition constants (*K_i_*) were calculated using Equation (4):

(4) $$\begin{equation*}{K_i} = \frac{{IC50}}{{1 + \frac{{{L_T}}}{{{K_D}}}}}\ \end{equation*}$$

where *L*_T_ is the concentration of the labelled peptide and *K_D_* is the dissociation constant of the labelled peptide F-pHDAC4 for MEF2A or MEF2D. All fluorescence polarization data were analysed using GraphPad Prism software.

### Glutathione *S*-transferase (GST) pulldown and co-immunoprecipitations

GST-MEF2D (1–190) was produced in BL21-DE3 competent cells as previously explained (15) and used as a bait. NIH-3T3 HDAC4/TM cells were lysed with a hypertonic buffer (25 mM Tris–HCl pH 7.5, 100 mM NaCl, 50 mM KCl, 5 mM MgCl_2_, 0.5% NP40, glycerol 10%, PIC 100×, PMSF 100×) Lysates were next incubated with 2 μg of GST-MEF2D and with HDAC inhibitors for 3 h at 4°C. For co-immunoprecipitation experiments, cells were lysed in a hypotonic buffer (20 mM Tris–HCl, pH 7.5; 2 mM EDTA; 10 mM MgCl_2_; 10 mM KCl; and 1% Triton X-100) supplemented with protease inhibitors. For each immunoprecipitation 1.5 μg of HDAC4 antibody or IgGs were used.

### RNA extraction and quantitative qRT-PCR

Cells were lysed using Tri-Reagent (Molecular Research Center). 1.0μg of total RNA was retro-transcribed by using 100 units of M-MLV Reverse transcriptase (Life Technologies) in the presence of 1.6 μM oligo(dT) and 4μM Random hexamers (Euroclone). qRT-PCRs were performed using SYBR green technology (KAPA Biosystems). Data were analyzed by comparative threshold cycle (delta delta Ct ΔΔCt) using *HPRT* and *GAPDH* as normalizer. The list of the primers used for qRT-PCR and ChIP-qPCR was previously published (19).

### RNA-seq analysis

SK-UT-1 cells were lysed using Tri Reagent (Molecular Research Center). Total RNA was treated with DNAse I (NEB) and purified with RNA Clean & Concentrator (Zymo Research). RNA-seq library preparation and sequencing were performed at BMR-Genomics (Padua, Italy) following Illumina specifications. Quality control for raw sequencing reads was performed with programs FastQC (v0.11.9) (www.bioinformatics.babraham.ac.uk/projects/fastqc/) and MultiQC (v1.09) (29). Transcript quantification was conducted with Salmon (v1.4.0) (30) on human transcriptome GRCh38 Ensembl version 100 (gene set patch level 13). Transcript quantifications were imported into R (v4.0.3) running Bioconductor (v3.11) for downstream analysis with tximeta (v1.6.3) (31) and summarized at the gene level. Principal component analysis was carried out with the plotPCA function from the DESeq2 package (v1.28.1) (32). Genes with a raw counts mean <64 between each condition replicates were removed from the analysis. Differential expression analysis was performed using DESeq2 with Wald test for significance. We adjusted for multiple hypothesis testing by employing Benjamini-Hochberg correction at a false discovery rate (FDR) of 0.05. Genes reported significantly by DESeq2 with an absolute fold change >2 were considered as differentially expressed. Genes were annotated with package AnnotationHub (v2.20.2) utilizing Ensembl annotation 100 data.

Normalization within differential expression analysis was run on the full dataset, including three samples treated with a derivative of NKL54 lacking the trifluoromethyl group (NKL22) (9). As only one time point was available for this inhibitor, NKL22 treatment was excluded from the downstream analyses. Plots were generated with ggplot2 (v3.3.3). Venn diagrams were created with VennDiagram (v1.6.20) or with the Venn diagram tool by the bioinformatics and evolutionary genomics group at VIB/Ghent University (http://bioinformatics.psb.ugent.be/webtools/Venn/). Functional annotation was performed on KEGG, Reactome slimGO and Gene Ontology databases with ClusterProfiler (v3.16.1) and ReactomePA (v1.32.0), respectively (19,33). GO term and pathway analysis results are reported at an FDR of 0.05.

### ChIP, library construction and ChIP-seq data analysis

Chromatin was obtained from SK-UT-1 cells, 14 h after DMSO or NKL54 (5μM) treatment and immunoprecipitated with 2 μg of anti-H3K27ac, 3 μg of anti-MEF2D antibody, 4 μg of anti-HDAC4 or anti-HDAC9 antibodies or control IgG, as previously described (34). Three independent biological replicates were pulled according to BLUEPRINT requirements and 5 ng of total DNA were used to prepare ChIP-seq libraries, according to TruSeq ChIP Sample Preparation guide (Illumina). Libraries were sequenced on the Illumina HiSeq 2000 sequencer. The ShortRead R/Bioconductor package was used to evaluate the quality of sequencing reads and Bowtie 2 was used to align them to NCBI GRCh38 human genome reference. Peak calling and gene annotations were performed as previously described (19). ChIP-seq replicates were compared using the Irreproducibility Discovery Rate (IDR) framework (35), with the MACS2 narrow peaks as input and applying the following settings: –input-file-type narrowPeak, –rank signal.value, –output-file-type narrowPeak. The IDR reported peaks were used for further processing. Gplots, BiomaRt, and Gviz R/Bioconductor packages and the DeepTools suite were used to generate peak heatmaps and for the visualization of genomic loci. DeepTools was also used for generating the correlogram showing the genome-wide Spearman correlation between the ChIP-seq replicates (average scores per genomic bin (10kb)).

### Statistics

For experimental data Student t-test was employed. Mann–Whitney test was applied when normality could not be assumed. We chose *P* < 0.05 as the statistical limit of significance. For comparisons between more than 2 samples, the Anova test was applied coupled to Kruskal–Wallis and Dunn's Multiple Comparison Test. We marked with * *P* < 0.05, ** *P* < 0.01, *** *P* < 0.001. Unless otherwise indicated, all the data in the figures were represented as arithmetic means ± the standard deviations from at least three independent experiments.

## RESULTS

### NKL54 induces cell death by apoptosis in LMS cells

LMS is a rare and aggressive tumor that has some smooth muscle features and accounts for ∼10% of adult STS (12,13). The therapeutic outlook for advanced LMS has not improved over the past decades, thus new approaches are urgently needed. The MEF2/Class IIa HDACs axis is dysregulated in a constant percentage of LMS. Furthermore, genetic ablation of this axis results in impaired cell growth with induction of cell death (19,36). These properties make this axis a promising therapeutic target for the treatment of LMS. For these reasons, we investigated previously characterized small molecules inhibitors of MEF2 class IIa HDACs interaction (BML-210 and NKL54) (9), for their ability to affect proliferation of LMS cells. BML-210 and NKL54 are pimelic diphenylamides belonging to the benzamide group. These compounds were initially identified and characterized as selective class I HDACs inhibitors (37).

BML-210 and NKL54 were compared for their ability to suppress LMS cell proliferation with the class IIa-selective HDAC inhibitor TMP195 and the pan-HDAC inhibitor SAHA (suberoylanilide hydroxamic acid), a zinc-chelator (Supplementary Figure S1A) (38,39). Two different LMS cell lines DMR and SK-UT-1 were used. SAHA showed the strongest antiproliferative activity with an IC_50_ of 2.7 μM in both cell lines (Supplementary Figure S1B/C). TMP195 only slightly affected the proliferation of LMS cells with an IC_50_ of 50 μM. NKL54 was more potent than BML-210 in both cell lines with an IC_50_ of 7.9 and 10.4 μM compared to the values of 20.2 and 18.3 μM of BML-210 (Supplementary Figure 1B/C). Next, we examined the induction of cell death. We chose NKL54 for its stronger antiproliferative activity compared to BML-210, and cell death was assessed by propidium iodide positivity and caspase activity using the DEVDase assay. Since the knock-out of HDAC9 in SK-UT-1 cells increases FAS expression and susceptibility to cell death, we evaluated the contribution of the extrinsic apoptotic pathway in NKL54-induced cell death. LMS cells expressing the inhibitor of DISC-activation FLIPs (the short isoform of CFLAR/FLIP) were used (40). Dose-dependent studies showed that NKL54 induces cell death in LMS cells, in part via the extrinsic pathway (Supplementary Figure 1D). Caspase activation demonstrates induction of apoptosis and dependence on the extrinsic pathway (Supplementary Figure 1E). Finally, NKL54 treatment increased FAS mRNA levels in LMS cells (Supplementary Figure 1F). In conclusion, NKL54 can induce apoptosis in LMS cells. This cell death response is characterized by the upregulation of FAS, as was also observed in cells null for HDAC9 (19).

### Identification and characterization of new PAOA derivatives

**Figure 1.**
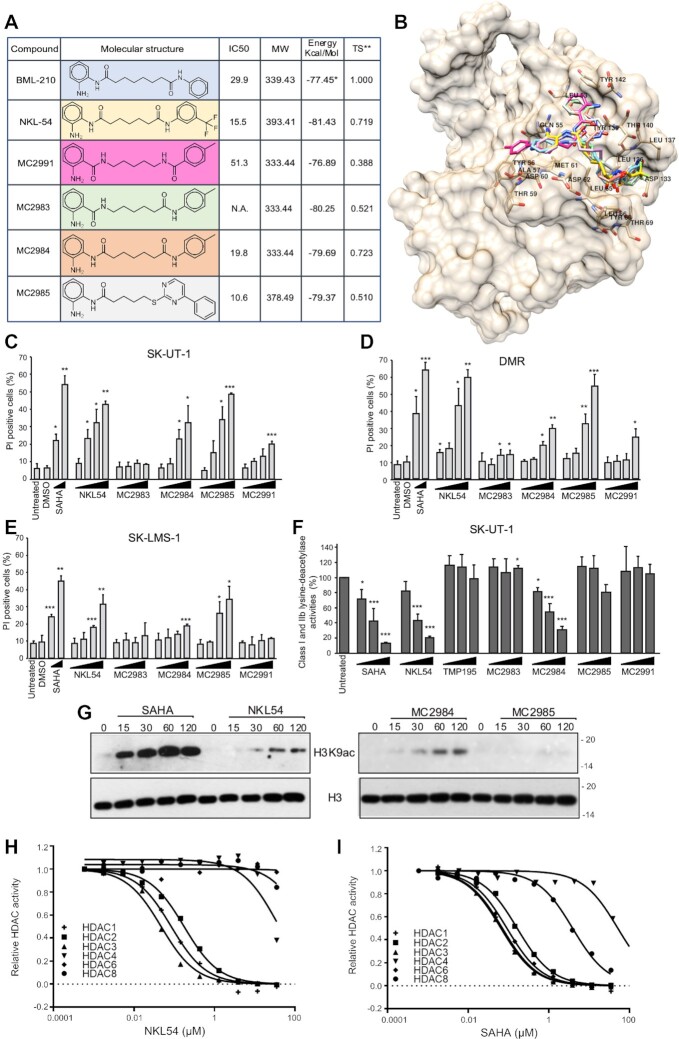
Characterization of new PAOA derivatives for mechanisms of action and anti-proliferative activities in LMS cells. (**A**) Characteristic and chemical structures of different PAOA derivatives used in this study in comparison with BML-210 and NKL54. The IC50 in SK-UT-1 cells is show as well as the Plants/PLP docking energies for the compound assayed in this study. The Tanimoto coefficient is reported for all the compounds versus the reference BML-210. *Energy calculated on the re-docked ligand extracted from the 3MU6 complex available from PDB. **Tanimoto similarity index calculated with radius 2 circular Morgan fingerprints (73) in a python script (74) using the RDKit library (75). (**B**) BML-210, NKL54, (MC2983, MC2984, MC2985 and MC2991 docked conformations in the MEF2A (pdb entry code 3MU6) hydrophobic groove. Plants/PLP combination as implemented in 3d-qsar.com was used to dock the compounds. Lowest energy docked conformations were imported in UCSF Chimera along with the cleaned minimized MEF2A protein and co-crystallized BML-210, for binding mode inspection and comparison. (**C**) Analysis of cell death in SK-UT-1 cells as percentage of PI positive cells treated with the indicated compounds. Cell death was scored after 48 h from treatments. Data are from three independent experiments, + S.D. (**D**) Analysis of cell death in DMR cells as percentage of PI positive cells treated with the indicated compounds. Cell death was scored after 48 h from treatments. Data are from three independent experiments, + S.D. (**E**) Analysis of cell death in SK-LMS-1 cells as percentage of PI positive cells treated with the indicated compounds. Cell death was scored after 48 h from treatments. Data are from 3 independent experiments, + S.D. (**F**) Lys-deacetylase activity as measured from SK-UT-1 cells treated with increasing concentrations of the indicated compounds [1, 5, 10 μM]. Data are from three independent experiments, + S.D. (**G**) Immunoblotting analysis of H3K9 acetylation levels in SK-UT-1 cells treated with SAHA [2.5 μM], NKL54, MC2984 and MC2985 [5μM] for the indicated minutes. (**H**) Dose response curves of NKL54 against a panel of indicated HDAC isozymes using standard enzyme activity assays. (**I**) Dose response curves of SAHA against a panel of indicated HDAC isozymes using standard enzyme activity assays.

To identify new compounds with improved ability to disrupt the interaction between MEF2 and class IIa HDACs, we synthesized a series of small molecules resembling NKL54 structure (MC2983, MC2984, MC2985 and MC2991), as shown in Figure 1A. MC2983 and MC2991, which present the amide group in an inverted position, should be less efficient as zinc chelators, but still able to interact with MEF2s similarly to the other compounds.

To confirm this hypothesis, a virtual screening was performed using the structure of MEF2A. The binding affinity of the different compounds to the hydrophobic groove of MEF2s was compared. For this purpose, the program PLANTS with the PLP scoring function was used (41). Docking energy analysis indicates that the new compounds should be able to interact with the hydrophobic groove of MEF2 with similar potency to NKL54 (Figure 1A and Supplementary Table S1). Indeed, NKL54 is estimated to be the most potent with a docking energy of −81.43 Kcal/mol. Among the four new compounds, MC2983 is predicted to be the most active, with a docking energy (−80.25 kcal/mol) slightly lower than that of NKL54. Interestingly, MC2991, the compound most divergent to the reference BML-210, is estimated to be the least potent of the series. In fact, MC2991 is the only compound that shows a docked conformation that does not fit the hydrophobic groove of MEF2 (Figure 1B and Supplementary Figure S2A).

Next, the different compounds were tested for cell death induction. Three different LMS cell lines were used: SK-UT-1, DMR and SK-LMS-1. SAHA is the reference for a pan-HDAC inhibitor and zinc chelator. The results were comparable in the three cell lines, with SK-LMS-1 cells showing some resistance to cell death, as observed previously (14). Compounds MC2983 and MC2991, which are structurally related to NKL54 but should not act as zinc chelators, were significantly much less effective in inducing cell death (Figure 1C–E).

The different ability of the tested compounds to inhibit class I and IIb HDACs was confirmed *in vivo*. Apart from MC2985, which inhibits lysine deacetylase activities only at high concentrations, all compounds capable of inducing cell death also inhibited KDACs (Figure 1F). As expected, MC2983 and MC2991 were inactive in this assay. SAHA and TMP195 were used as positive and negative controls, respectively.

To confirm the ability of NKL54, MC2984 and MC2985 to act as epigenetic drugs, we examined the levels of histone H3 lysine 9 acetylation (H3K9ac) by immunoblot. SAHA triggered an increase in H3K9 acetylation within 15 min of treatment (Figure 1G). NKL54 and MC2984 also increased H3K9 acetylation, albeit with lower efficiency, compared with SAHA. The increase in H3K9ac in response to MC2985 treatment was very small, almost undetectable. All these compounds and BML-210 also increased H3K27ac levels with similar kinetics and potency as for H3K9ac (Supplementary Figure S2B). Overall, the modulation of H3K9ac and of H3K27ac levels in response to the different compounds confirms the inhibitory potency observed with the KDAC assay *in vivo*. Finally, we compared the *in vitro* inhibitory activity of SAHA and of PAOA derivatives against different purified HDACs (NKL54 was chosen as an example). The inhibitory activity of NKL54 was specific to HDAC1/2/3 (Figure 1H). SAHA confirmed the broader effect by inhibiting HDAC1/2/3/6 and with lower potency also HDAC8 (Figure 1I). IC50s are shown in Supplementary Table S2. Curiously, NKL54 can inhibit HDAC4 at high concentrations (IC_50_ 35μM), possibly due to the trifluoro group (42).

### The PAOA derivatives do not unleash the interaction between MEF2 and class IIa HDACs

**Figure 2.**
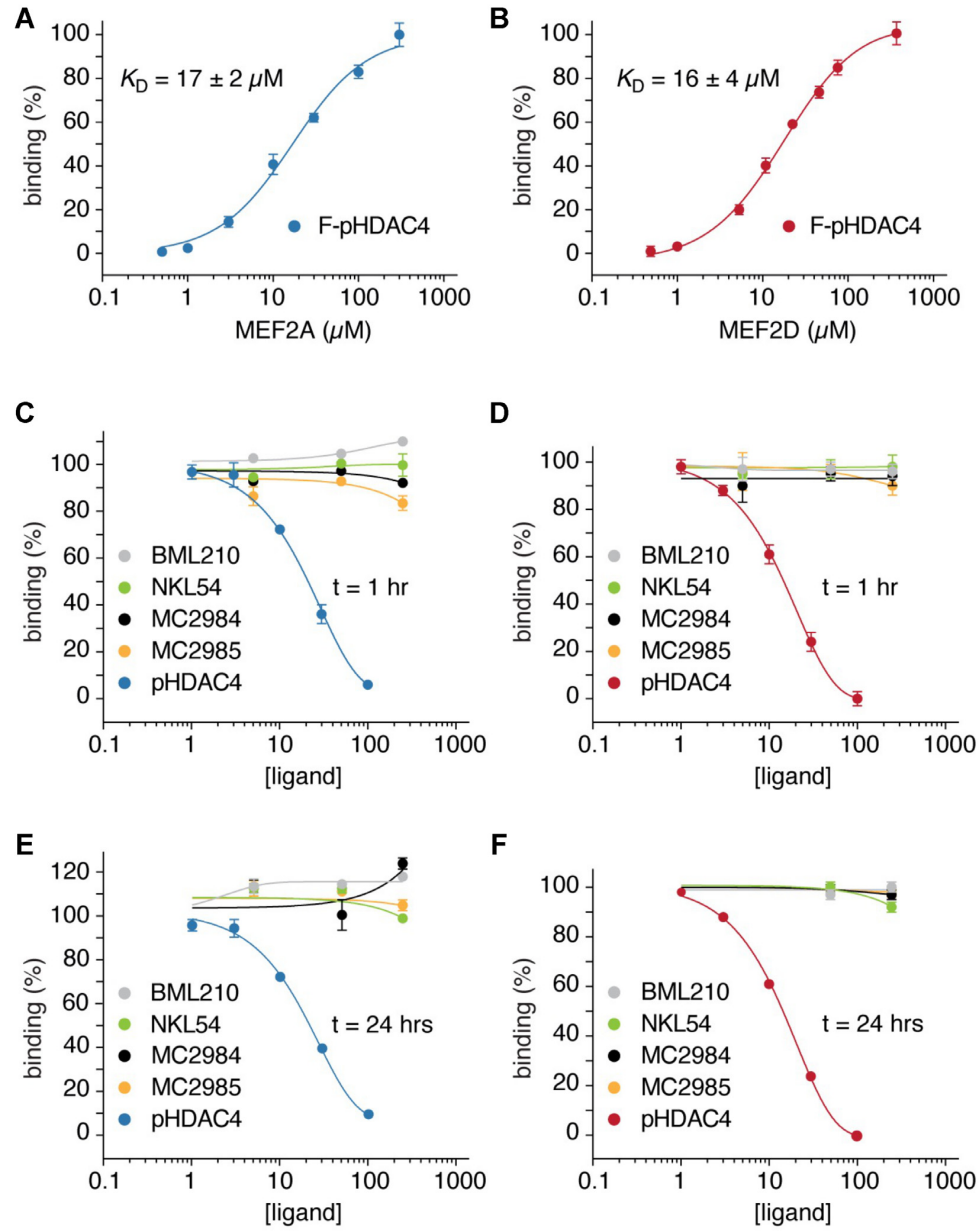
NKL54 does not compete for the binding between MEF2 and class IIa HDACs. (**A**) MEF2A binding curve (0.5–300 μM) titrated to fluorescently labelled peptide F-pHDAC4 (0.26 μM). The average fluorescence polarization values of at least three independent experiments ± SD were plotted as a function of ligand concentration. (**B**) MEF2D binding curve. Experiments were performed as in panel A. (**C**) Protein binding by the indicated compounds was measured by incubating different concentrations of each ligand (5-fold dilutions, ranging from 10 to 250 μM) with 0.26 μM F-pHDAC4 and 20 μM of MEF2A. Unlabelled pHDAC4 peptide (3-fold dilutions ranging from 1 to 100 μM) was used as positive control. Titrations were performed at room temperature for 1 h as indicated. The average fluorescence polarization values of at least 3 independent experiments ± SD were plotted as a function of ligand concentration. (**D**) MEF2D binding by the indicated compounds as performed in panel C. (**E**) MEF2A binding by the indicated compounds as performed in panel C, with titrations measured at 24 h. (**F**) MEF2A binding by the indicated compounds as performed in panel E.

**Figure 3.**
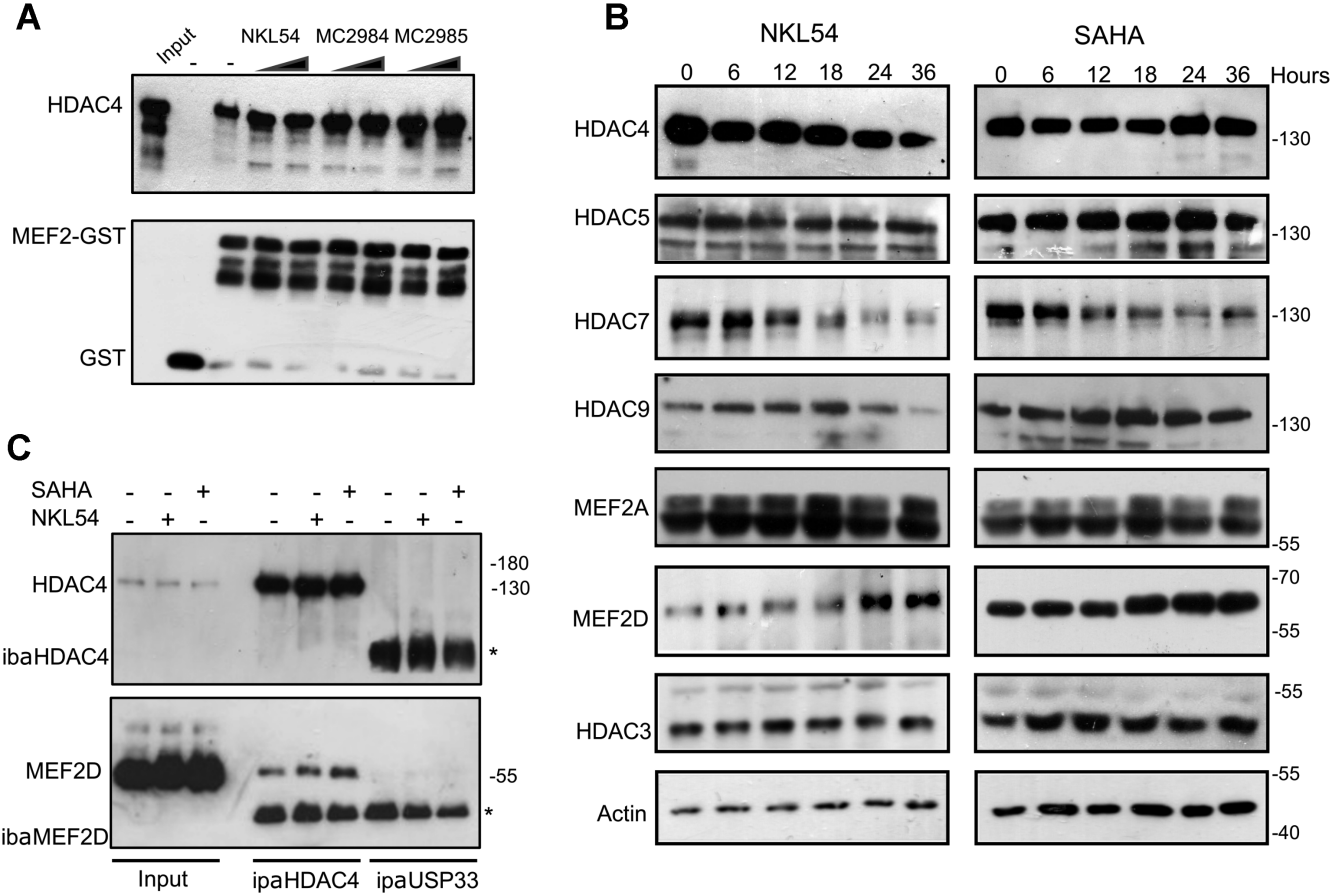
Regulation of MEF2-class IIa HDACs axis by PAOA-derivatives. (**A**) GST pull-down assay, using recombinant MEF2D (1–190) or GST as control. Purified GST or GST-MEF2D recombinant proteins (2 μg) were incubated with cellular lysates obtained from NIH3T3 cells overexpressing HDAC4 mutated in 14–3–3 binding sites. Two different concentrations [14 and 42 μM] were used. Immunoblots were performed to visualize HDAC4 or recombinant GST. (**B**) SK-UT-1 cells were treated for the indicated times with NKL54 [5 μM] or SAHA [2.5 μM]. Cellular lysates were generated and immunoblot performed using the indicated antibodies. Actin and Histone 3 (H3) were used as loading control. (**C**) Lysates from SK-UT-1 cells treated for 12 h with DMSO or with SAHA [2.5 μM] or NKL54 [5μM] MEF2D-HDAC4 complexes were immunoprecipitated with antibodies against HDAC4 or USP33, as a control. Immunoblotting using an anti-MEF2D antibody was next used for the detection of the MEF2-HDAC4 complexes. Asterisks point to IGs.

We have shown that the PAOA derivatives, that induce cell death in LMS cells, can act as HDAC inhibitors. However, the same derivatives could also affect the action of MEF2 by binding its hydrophobic groove and abolishing the inhibitory influence of class IIa HDACs. To verify the possible dual action of these compounds, we set up an *in vitro* fluorescence polarization assay (FP). By using a fluorescent labelled HDAC4-derived peptide (F-pHDAC4), capable of binding the hydrophobic groove of MEF2, we could assess the ability of the different compounds to unleash the interaction between MEF2 and class IIa HDACs. Recombinant MEF2A and MEF2D were produced and purified to homogeneity. First, the ability of the fluorescent labelled HDAC4-derived peptide (F-pHDAC4) to bind the MEF2 proteins was tested using a direct FP binding assay. MEF2A and MEF2D show similar affinity for pHDAC4 with a *K*_D_ of 16–17 μM (Figure 2A and B). Next, a FP competition assay was used to assess the ability of the different compounds to disrupt the interaction between MEF2A with F-pHDAC4 (Figure 2C). The same assay was performed with MEF2D (Figure 2D). We used an unlabelled HDAC4 peptide (pHDAC4) as a positive control. Although the affinity of the F-pHDAC4 probe does not allow to accurately assign compounds with binding affinity below 16μM, none of the tested PAOA derivatives was able to displace F-pHDAC4 from MEF2A or MEF2D, even when tested at concentrations ten times higher (250μM) the affinity of the probe. In contrast, pHDAC4 efficiently competed with F-pHDAC4 for MEF2A or MEF2D binding, and pHDAC4 released F-pHDAC4 within 1 h, indicating that binding was dynamic. We also investigated whether the PAOA derivatives could compete with F-pHADC4 during prolonged incubation times. However, longer incubation (24 h) did not disrupt the interaction between MEF2A or MEF2D with F-pHADC4 (Figure 2E and F). The inability of these compounds to compete for the binding of MEF2 with class IIa HDACs was also verified by a GST pull-down assay with recombinant MEF2D and HDAC4-GFP. Recombinant GST-MEF2D (2 μg) was incubated with cell lysates from NIH3T3 cells overexpressing HDAC4 with mutations in the 14–3–3 binding sites. This mutant cannot be phosphorylated and exported from the nucleus, increasing the pool of HDAC4 available for MEF2D binding (Figure 3A and Supplementary Figure S2C). Finally, immunofluorescence analysis confirmed that PAOA derivatives cannot interfere with the ability of MEF2D to cause nuclear accumulation of HDAC4 (Supplementary Figure S3).

### NKL54 and SAHA influence the expression levels of MEF2D and HDAC7

Pan-HDAC inhibitors such as hydroxamates (TSA or SAHA) and benzamides (entinostat and mocetinostat) affect the stability of HDAC7 (43). Consequently, these compounds might indirectly upregulate MEF2-dependent transcription by reducing the expression of class IIa HDACs. To verify this hypothesis, we analyzed the expression levels of the different members of the class IIa HDACs family in SK-UT-1 cells in response to treatment with SAHA or NKL54. NKL54 was chosen as a prototype for the different PAOA derivatives. We also analyzed the levels of MEF2A and MEF2D, which are the two major MEF2 family members expressed in this cell line. Figure 3B shows that HDAC7 is downregulated starting 12 h after addition of SAHA. Similarly, NKL54 triggers the downregulation of HDAC7. HDAC4 and HDAC9 are also downregulated at later time points (starting from 24 h), but only in NKL54-treated cells. In contrast, MEF2D expression levels were increased after treatment with the two HDACIs. These results were confirmed by a second immunoblot analysis and relative densitometric evaluations (Supplementary Figure S4A/B). When used at high concentrations, MC2984 and MC2985 also upregulated MEF2D expression and reduced HDAC7 levels (Supplementary Figure S2D).

This detailed characterization allowed us to select the best time point to verify the ineffectiveness of NKL54 in releasing the binding between MEF2D and HDAC4 *in vivo* as well. SK-UT-1 cells were treated with NKL54 or SAHA, as control, and 12 h later cellular extracts were generated and subjected to co-immunoprecipitation with an anti-HDAC4 antibody (Figure 3CSupplementary Figure S2C). This time point was chosen to preclude excessive variation in protein concentrations of the two targets. Consistent with the *in vitro* studies, NKL54 was ineffective in releasing MEF2D from HDAC4 binding *in vivo*.

### Transcriptome remodelling as elicited by different HDAC inhibitors

**Figure 4.**
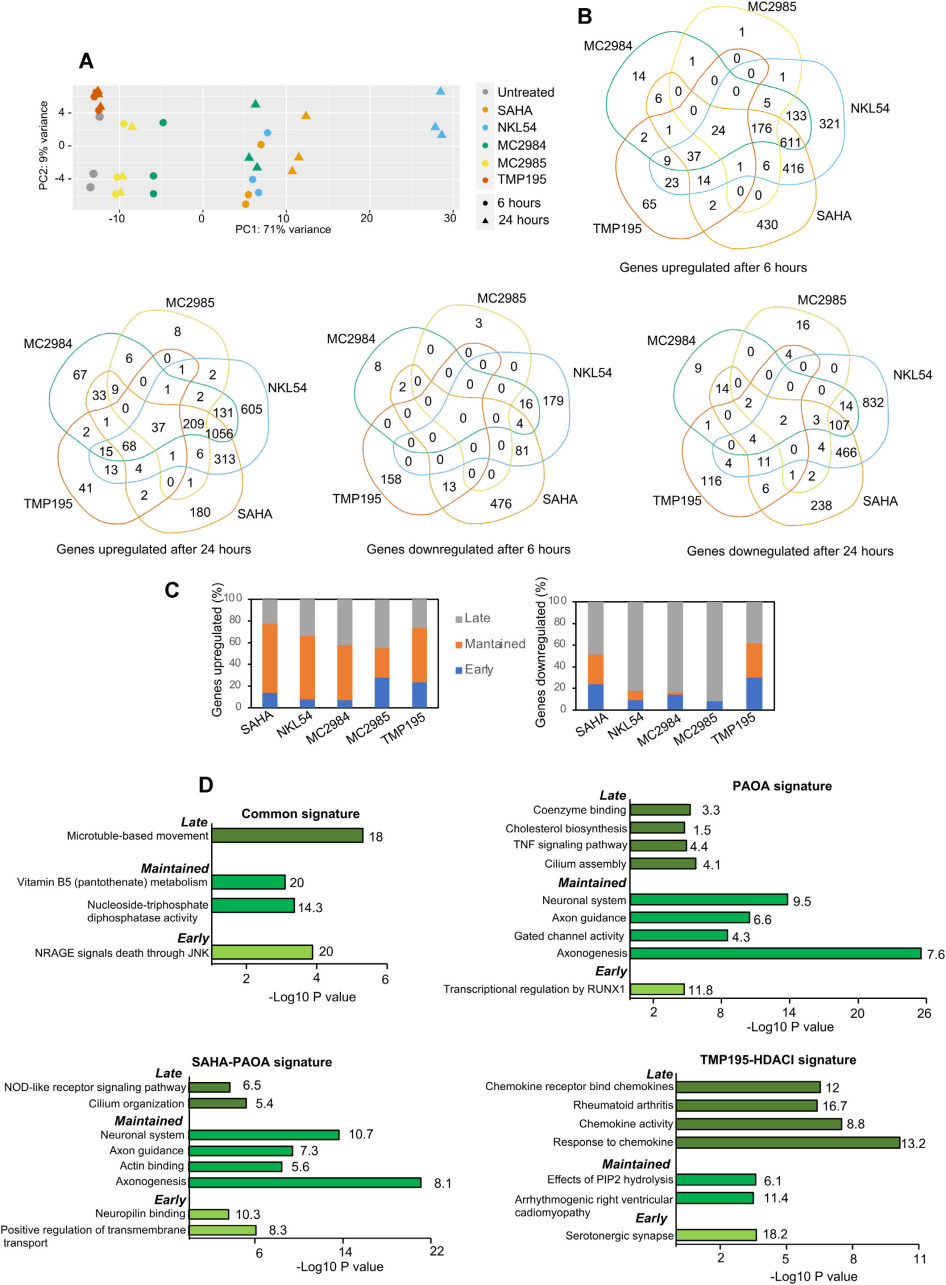
Comparative transcriptomic analysis in response to the different HDACIs. (**A**) PCA analysis performed on the expression profiles of the indicated treatments at shown times in SK-UT-1 cells. (**B**) Venn diagrams showing the number of transcripts commonly and differentially upregulated or downregulated between the different HDACIs at the indicated hours. (**C**) Percentage of genes upregulated or downregulated by the different HDACIs at 6 h (early genes) at both 6 and 24 h (maintained genes) and at 24 h (late genes). (**D**) Bar plots of the ClusterProfiler-ReactomePA most significantly enriched functional terms according to the GO: Biological Process, GO: Molecular Function, Reactome or KEGG databases. The analysis was performed for the indicated groups of signatures, retaining the top terms for each functional database. Numbers to the right of the bars represent the percentage of significantly enriched genes found within each category.

To better classify the different HDACIs, the induced transcriptional changes were mapped by RNA-seq. A single concentration of the different compounds (2,5μM for SAHA and 5μM for all PAOA-derivatives), triggering moderate and comparable percentages of cell death at 36 h was used. Percentages of cell death were: 23.2%*±*1.92 for SAHA, 21.4% ± 3.44 for MC2984, 27.0% ± 4.0 for MC2985 and 39.00% ± 2,44 for NKL54. In untreated cells the percentage of cell death was 7.00%± 2.24. We also used TMP195 (5 μM) a poor inducer of cell death (13.0% ± 1.0). RNA was isolated at 6 h, to map early changes, and after 24 h from treatments, to investigate delayed modulations in gene expression. The PCA (principal component analysis) shows the high reproducibility of the three biological replicates analyzed (Figure 4A). TMP195 induced few modifications in the transcriptome. Twenty four hours of treatment with MC2984 elicited modifications of the transcriptome like the early responses to SAHA and NKL54 treatments, thus suggesting a similar but delayed effect of this PAOA-derivatives. Finally, later changes in gene expression more manifestly separate NKL54 from SAHA (Figure 4A). The top upregulated and downregulated genes for each treatment are listed in Supplementary Tables S3 and S4.

To further validate these observations, we compared genes upregulated and downregulated in response to the different compounds using SAHA as a reference. Venn diagrams show that 66.8% of genes regulated at 6 h by NKL54 are shared with SAHA (Supplementary Figure S5). After 24 h 82.5% of genes modulated by SAHA were similarly modulated by NKL54. In parallel, the number of NKL54 specific genes increased up to 41.3%. Only 19.4% of genes (*n* = 184) were specific for MC2984 at 6 h, a percentage that decreased to 13.7% after 24 h of treatment (*n* = 246). MC2985 modulated very few genes at both 6 and 24 h (*n* = 218 and 317, respectively), of which only 4.6% (6 h) and 9.5% (24 h) are specific. TMP195 showed the most divergent profile respect to SAHA with only 48.4% of genes in common at 6 h. Interestingly, this percentage increased up to 60,5% after 24 h of treatment, thus indicating common adaptive responses (Supplementary Figure S5).

SAHA, NKL54 and MC2984 upregulated a larger number of genes compared to TMP195 and MC2985 (Figure 4B and Supplementary Figures S5 and S6A). For SAHA and NKL54 the effect on gene transcription was rather stable through the time, with respectively 64, and 61% of genes upregulated at both time points (Figure 4C). There are also genes which expression was upregulated at 24 h but not at 6 h. The percentage of these genes was lower for SAHA (22.6%) and TMP195 (26.4%), intermediate for NKL54 (33.6%) and greater for MC2984 and MC2985 (42 and 45%, respectively). These different delayed responses could reflect different kinetics/modifications of the compounds or different adaptive responses engaged. Among the early DEGs (differentially expressed genes), the downregulated genes were a minor fraction (Supplementary Figure S6): 13.6% (NKL54), 2.8% (MC2984), 1.5% (MC2985) and 25% (SAHA).

Again, the response to TMP195 was highly divergent, with 45.9% of DEGs that were downregulated. Interestingly, in all treatments, the down-regulated genes were modulated in higher percentages at 24 h (Figure 4C and Supplementary Figure S6B). This feature is particularly evident for the PAOA derivatives. Here, the few overlaps observed between 6 and 24 h suggest that many genes downregulated at later time points may be indirect targets of these compounds.

### Common biological responses modulated by the different HDACIs

The above analysis has shown that the various inhibitors trigger both common and specific transcriptional modulations. To define the respective cellular responses, we selected both compound-specific DEGs and DEGs common among different compounds. Common genes were grouped into three distinct categories: (i) the SAHA-PAOA signature, which includes genes regulated by SAHA and also by at least one PAOA derivative; (ii) the PAOA signature, which includes genes regulated by at least two different PAOA derivatives; and finally, (iii) the TMP195-HDACIs signature, which includes genes regulated by TMP195 and also by at least SAHA or a PAOA derivative. Gene signatures were also analyzed in terms of temporal regulation (Supplementary Figure S7A and B). The early response includes genes regulated at 6 but not at 24 h of treatments. The maintained response includes genes regulated at both 6 and 24 h Finally, the late response includes genes that are regulated at 24 h, but not at 6 h.

We used ClusterProfiler (33) and ReactomePA (44) to understand the functions of genes that are regulated under the different treatments. First, we evaluated upregulated genes. The top category of genes that are upregulated by all inhibitors is related to microtubules-based movements and represents a late response (Figure 4D and Supplementary Table S3). For the SAHA-PAOA signature, neuronal system, axon guidance, and axonogenesis are the most significant results. Since these genes belong to the maintained category, this response might reflect the stable release of a cell-lineage specific inhibitory influence of HDACs. These gene categories are also dominant for the PAOA signature. For both the SAHA-PAOA and PAOA signatures, early and late responses are less clearly defined and include both metabolic and differentiative responses. The TMP195-HDACIs signature is characterized by the chemokine activity/rheumatoid arthritis categories which included the late-responding genes (Figure 4D and Supplementary Table S5).

We also examined cellular responses that were switched off after HDACIs treatment. As expected from the general trends of downregulation (Supplementary Figure S7B), the most significant enrichments were found within the late responses. Interestingly, in the SAHA-PAOA and PAOA signature the most enriched categories were found in the context of chromatin organization (HAT acetylate histones, DNA packaging, Nucleosome assembly, DNA conformation change) possibly reflecting a compensatory response triggered by changes in chromatin dynamics, because of HDACs inhibition (Supplementary Figure S7C and Supplementary Table S6). Another, highly enriched downregulated category is M phase, a plausible consequence of cell-cycle arrest. As observed above for the upregulated genes, the TMP195-HDACIs categories show again specific features with an impact on the microenvironment. Indeed, the ECM genes are among the most highly enriched (Supplementary Figure S7C and Supplementary Table S6).

### Specific biological responses engaged by the different HDACIs

Compound-specific DEGs represent a small percentage of regulated genes (Figure 4B). As described above for common DEGs, these signatures were analyzed in relation to the timing of regulation by dividing the DEGs into early, maintained, and late. Among the upregulated genes, significant categories were found for NKL54, SAHA, and TMP195 (Supplementary Figure S8A, Supplementary Table S7). The biological processes diverge greatly among the three compounds. SAHA derepresses genes related to cilia/flagellar organization and dynamics (cilium assembly organization, intraflagellar motility) as maintained and late response. NKL54 affects extracellular matrix dynamics, differentiation and metabolism as an early, maintained and late response. TMP195 shows early activation of chemokine expression and inflammatory responses that are later induced by the other HDACIs (Figure 4D). These selective influences on gene expression are indicative of the existence of distinct complexes containing class I or class IIa HDACs.

Among the down-regulated genes, again NKL54, SAHA and TMP195 achieve the most significant enrichments (Supplementary Figure S8B, Supplementary Table S8). Genes involved in the maintenance of chromatin homeostasis are strongly repressed by both SAHA and NKL54, as observed above for some common signatures. In addition, NKL54 significantly represses cell cycle-related genes. TMP195 shows a differential effect on the ECM, the microenvironment as an early response and, both as an early and late response, on some genes controlling differentiating responses.

### SAHA and PAOA derivatives boost the expression of BH3-only BCL2 family members

**Figure 5.**
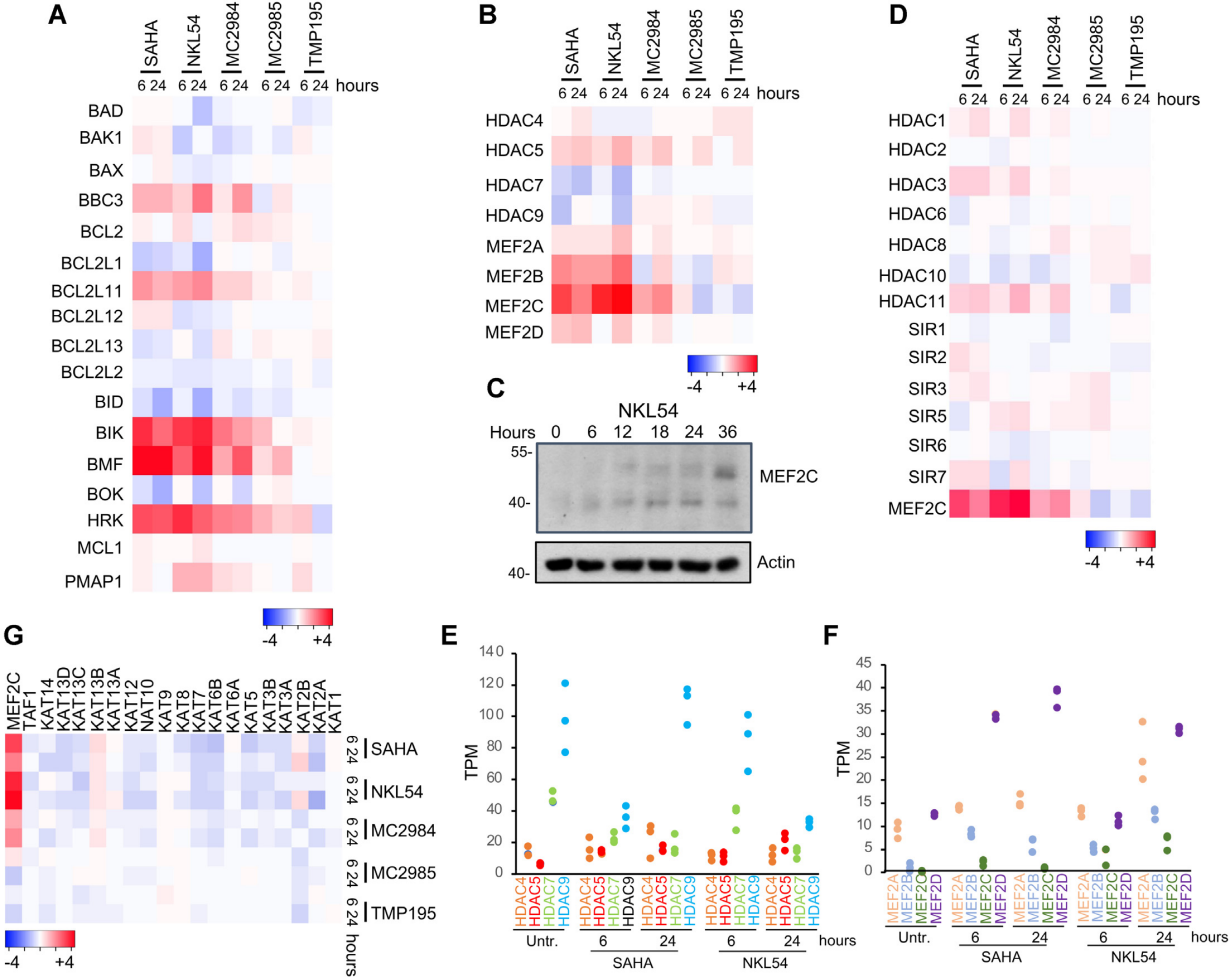
Influence of the different HDACIs on the expression levels of BCL2 family members, of the MEF2-HDAC axis and on KATs. (**A**) Heat-map reporting the expression levels (log_2_ fold change relative to untreated cells) of the indicated BCL2 family members in response to the different HDACIs as indicated. (**B**) Like panel A, instead showing MEF2-HDAC axis components. (**C**) SK-UT-1 cells were treated for the indicated times with NKL54 [5μM]. Cellular lysates were generated and immunoblot performed using the anti-MEF2C antibody. Actin was used as loading control. (**D**) Heat-map reporting the expression levels (log2 fold change relative to untreated cells) of class I/IIb/III/IV HDACs family members in response to the different HDACIs as indicated. (**E**) Heat-map reporting the expression levels (log_2_ fold change relative to untreated cells) of different KATs in response to the different HDACIs as indicated. (**F**) Class IIa HDAC family members expression in SK-UT-1 cells treated or not with SAHA and NKL54 for the indicated times. Expression values are shown in TPM (transcripts per million) calculated from a gene model where isoforms were collapsed to a single gene. (**G**) Like panel F, instead showing MEF2 family members.

Induction of cell death by apoptosis characterizes the response to SAHA and PAOA-derivatives. BCL2 family members are master regulators of apoptosis, through control of mitochondrial outer membrane permeabilization (45). Frequently cell death signalling pathways control the expression of BCL-2 family members. All of the different compounds that trigger cell death in LMS affect expression of a group of BH3-only members. mRNA levels of *BMF*, *BIK* and *HRK* are dramatically increased in response to SAHA and PAOA (although MC2985 was less potent) (Figure 5A). *BBC3/PUMA* and *BCL2L11/BIM*, other BH3-only members, are less upregulated. TMP195, which is a very weak inducer of cell death only modestly and transiently increases *HRK* and *PMAP1/NOXA* mRNA levels.

### SAHA and PAOA derivatives modulate the expression of members of the MEF2-HDAC axis

NKL54 and SAHA could indirectly affect the MEF2-HDAC axis by modulating the levels of MEF2 and HDAC family members (Figure 3B). Analysis of RNA-seq data showed that HDAC5 mRNA is slightly upregulated, whereas HDAC7 and HDAC9 are down-regulated (Figure 5B). Importantly, NKL54 exerts a persistent effect on HDAC9, whereas SAHA only transiently downregulates it. Accordingly, HDAC9 protein levels are significantly downregulated only in cells treated with NKL54 (Figure 3B). All other compounds only moderately affect the expression of class IIa HDACs.

When class IIa HDACs are generally downregulated in response to SAHA and NKL54, MEF2 family members are upregulated, particularly MEF2B and MEF2C (Figure 5B and C). The expression of other HDACs is only slightly affected by the investigated compounds. SAHA and the PAOA derivatives (NKL54 and MC2984) upregulate the mRNA levels of HDAC11, HDAC3, and to some extent HDAC1 (Figure 5D). To understand the impact of these regulations on the transcriptional activity of the MEF2-HDAC axis, we evaluated the TPM (transcript per million) measure for each family member. HDAC9 and HDAC7 yield the most highly expressed family members in SK-UT-1 cells (Figure 5E). Therefore, their down-regulation could strongly affect the total pool of class IIa HDACs available for suppression of MEF2 transcription. On the other hand, although MEF2C is strongly upregulated by SAHA, NKL54 and MC2984, because it is expressed at very low levels in SK-UT-1 cells, its impact on the overall MEF2 transcriptional output might be minimal (Figure 5C and F). In contrast, MEF2D is the most expressed paralog in this cell line (Figure 5C), and although it is much less upregulated compared with MEF2C, it could strongly influence the transcriptional landscape, as confirmed by the consistent increased protein levels (Figure 3B).

### The expression of several lysine-acetyltransferases (KATs) is downregulated after inhibition of HDACs

A prominent response to HDACIs is the downregulation of genes encoding components of the KAT complexes, an evolutionary adaptive mechanism for buffering HDACs inhibition (46). Therefore, we examined the expression levels of various KATs in response to the treatments (47). With few exceptions, SAHA/NKL54/MC2984 trigger downregulation of several KATs. MC2985 and TMP195 are much less effective and, curiously, both promote downregulation of KAT2B (Figure 5G). In general, there is a strong correlation between the ability of the various compounds to increase histone acetylation and the repression of KAT expression.

### NKL54 and the regulation of the MEF2-HDAC axis

**Figure 6.**
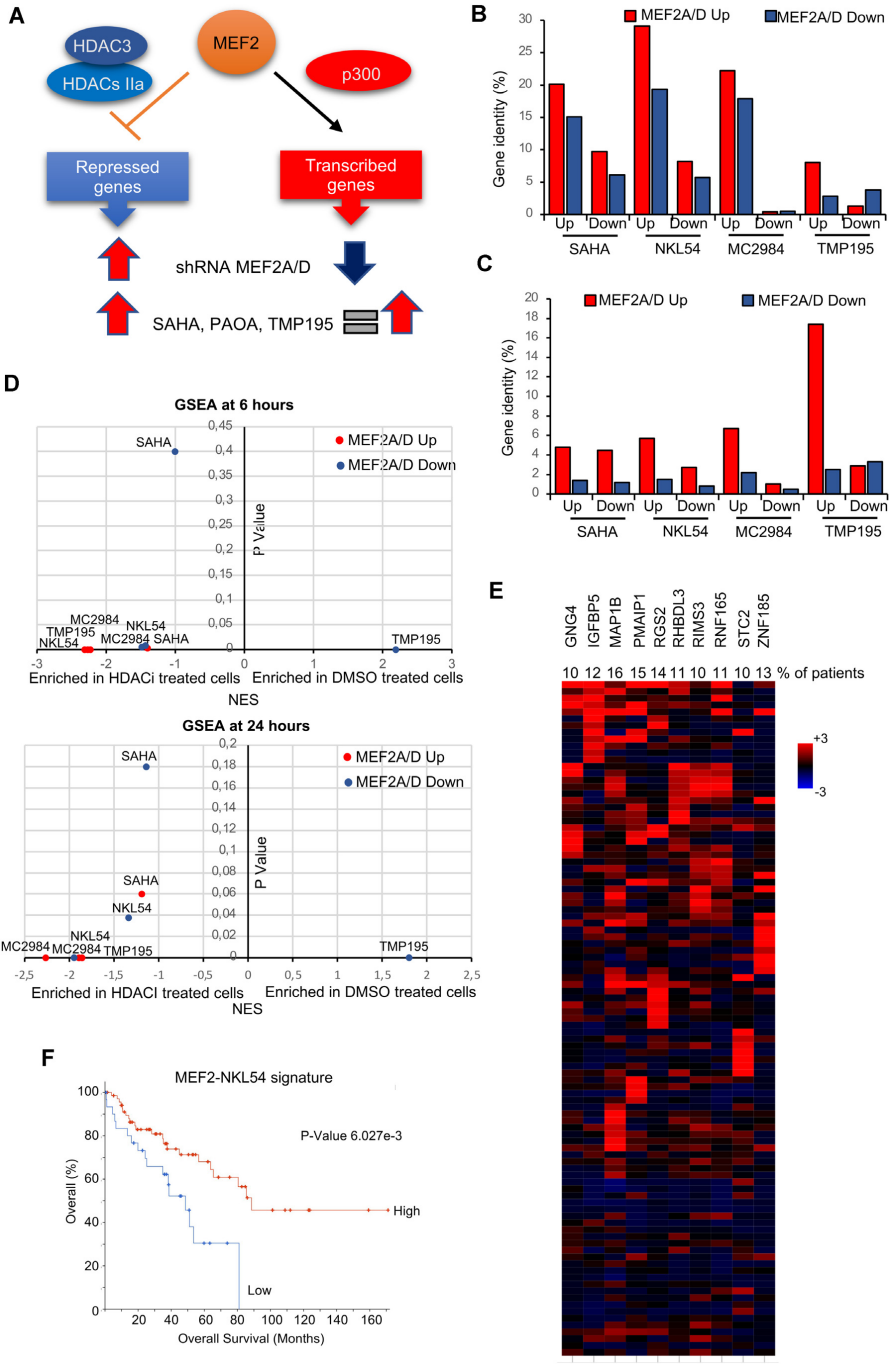
The impact of the different HDACIs on the MEF2 transcriptional activity. (**A**) Scheme comparing the effect of MEF2A/D silencing and HDACIs treatments on genes regulated by MEF2. (**B**) Percentage of identity among genes up or downregulated after MEF2A or MEF2D silencing and genes up or downregulated after treatments with the different HDACIs. (**C**) Percentage of identity among genes up or downregulated after treatments with the different HDACIs and genes up or downregulated after MEF2A or MEF2D silencing. (**D**) GSEA results displayed as the NES and the p value obtained by interrogating the transcriptome of MEF2A/D knocked-down SK-UT-1 cells with the transcriptomes of the same cells treated with the different HDACIs. (**E**) Oncoprint of mRNA expression variations for the indicated genes defined as MEF2-NKL54 signature. Data were obtained from the TCGA database and include RNAseq data of 100 patients with LMS. The heatmap shows the expression levels (z-score normalized log2 (FPKM) values) relative to diploid samples and was generated through cBioPortal (http://www.cbioportal.org). The percentages refer to the number of patients with *z*-score >2. (**F**) Kaplan–Meier plot showing the survival in percent of patients having expression of the ‘MEF2-NKL54’ gene signature (panel E) with at least one member with *z*-score >2 with respect to diploid samples, as represented by Kaplan–Meier plot. The graph was generated through cBioPortal (http://www.cbioportal.org).

NKL54 is not efficient in impeding the binding between MEF2 and class IIa HDACs, but could indirectly sustain MEF2-dependent transcription, through its influence on class IIa HDACs and MEF2D levels. MEF2A and MEF2D are the two major paralogs expressed in SK-UT-1 cells. Hence, we compared genes that were up and downregulated after silencing of these two MEF2 family members, that we defined previously (35), with genes that were modulated by the HDACIs under study. This comparison aimed to provide insight into the ability of NKL54 to affect the MEF2-HDAC axis. It is important to emphasize that in SK-UT-1 cells MEF2 act as transcriptional repressors at some loci and as transcriptional activators at others (35). Taking this into account, genes upregulated after treatment with the different compounds should be compared with genes upregulated after MEF2A/D silencing (abrogation of repression), and with genes repressed after MEF2A/D silencing (Figure 6A). In this last condition, the increased expression in response to HDACIs may depend on the augmented MEF2D levels. Certainly, the contribution of other TFs whose activities may be modulated by the different HDACIs cannot be excluded.

Venn analysis shows that MEF2-regulated genes have the best overlap with NKL54-regulated genes (Figure 6B and Supplementary Figure S9A). 29,1% of genes upregulated, and 19,3% of genes downregulated after MEF2A/D silencing are upregulated in response to NKL54. The other PAOA derivative (MC2984) is the second-best compound. 22,2% of up and 17,9% of downregulated genes after MEF2A/D silencing are upregulated by MC2984. Next, we examined the percentage of overlap between the genes regulated by the different compounds and the MEF2A/D signatures (Figure 6C and Supplementary Figure S9B). In this case, the best result was obtained for TMP195 with 17.4% identity among the up-regulated genes and only 2.5% among the down-regulated genes. This result is another confirmation of the existence of repressive MEF2 complexes in SK -UT-1 cells (35). In summary, this analysis shows that a small percentage of genes upregulated by NKL54, which has the broadest effect among the HDACIs tested, are under the regulation of MEF2A/D. In contrast, TMP195 has limited effects on overall gene expression, but a certain percentage of genes upregulated by this class IIa inhibitor are targets of the MEF2-HDAC axis. GSEA was applied to confirm these results. Again, the PAOA derivatives (NKL54 and MC2984) achieved the best enrichments compared with MEF2A/D signatures (Figure 6D).

From a therapeutic perspective, it is important to understand whether reactivation of the MEF2A/D signature could be beneficial for LMS patients. Therefore, we investigated whether genes co-regulated by MEF2A/D and NKL54 might be important for LMS aggressiveness. We selected genes modulated by MEF2A/D and upregulated in LMS cells after NKL54 treatment. From this signature, which includes 123 genes, we selected genes that were upregulated in at least 10% of patients (Figure 6E, *n* = 10). We defined this group of genes as the MEF2-NKL54 signature. Interestingly, the BH3-only member *PMAIP1/NOXA* is included in this signature. Figure 6F shows that patients characterized by high expression of genes of the MEF2-NKL54 signature have a better prognosis.

### NKL54-induced changes in H3K27ac genomic distribution

**Figure 7.**
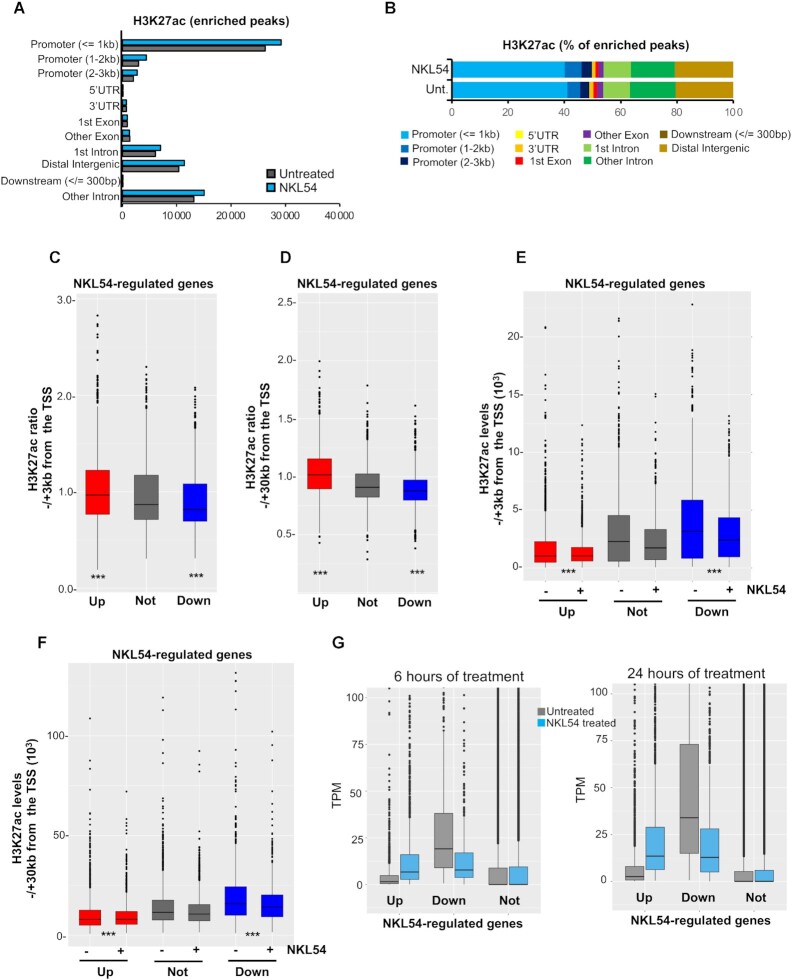
Variation of H3K27ac distribution at the genomic level. (**A**) Genomic distribution of the H3K27ac-enriched IDR-defined peaks identified by MACS2 in SK-UT-1 cells treated (*n* = 72 765) or untreated (*n* = 64 232) for 14 h with 5 μM NKL54. (**B**) As in panel A, with values represented as percentages. (**C**) H3K27ac ratio between NKL54 treated and untreated cells within ±3 kb from TSS. ChIP-seq data are from experiment 1. Genes not-regulated by NKL54 were selected based on having the lowest combined gene expression variations at 6 and 24 h from treatment. The boxes indicate the interquartile range with the center line representing the median value. The outliers are plotted as dots. (**D**) As in panel C, with H3K27ac ratio between NKL54 treated and untreated cells calculated within ±30 kb from TSS. (**E**) Overall acetylation level in the ± 3kb region centered on gene TSS of the indicated gene categories in presence or absence of NKL54. Boxes plotted as in panel C. (**F**) As in panel E with overall acetylation level measured within a ±30 kb region centered on gene TSS of the indicated gene categories in presence or not of NKL54. (**G**) TPM values are shown after treatment or not with NKL54 for the respective times. TPM measure was calculated from a gene model where isoforms were collapsed into a single gene. Significances were tested using the Mann–Whitney *U* test.

To better characterize the effect of NKL54 at the genomic level and its influence on gene expression, we examined the genomic distribution of H3K27ac by performing ChIP-seq. Two distinct sequencing experiments were done, each pooling at least two distinct biological replicates. We selected 14 h after treatment to limit the effect of NKL54 on the levels of class IIa HDACs. A Pearson's correlation coefficient (PCC) test on the two sequencing experiments was performed to assess the reproducibility of each ChIP-seq (48). The results were represented as a heatmap and show a very high reproducibility (Supplementary Figure S10). Treatment with NKL54 resulted in a higher number of IDR-defined H3K27ac peaks (*n* = 72 765) compared to control (*n* = 64 232). These peaks were particularly abundant in promoter regions near TSS and at distal intergenic regions (Figure 7A).

Experiments were done 14 h after treatment to limit the effect of NKL54 on the levels of class IIa HDACs. Treatment with NKL54 resulted in a higher number of H3K27ac peaks (*n* = 110 900) compared to control (*n* = 83 419). These peaks were particularly abundant in promoter regions near TSS and at distal intergenic regions (Figure 7A). HDACIs increased the number of enriched peaks in all genomic regions, with a slightly higher percentage in promoter regions within 1–3 kb from TSS (Figure 7B). Next, we focused the analysis on genes regulated by NKL54. For reference, we selected 2000 genes whose expression was not regulated by NKL54. Genes upregulated in response to NKL54 show an increase in H3K27ac around TSS (±3 kb) compared to those not regulated. In contrast, NKL54-downregulated genes show a reduction in H3K27ac levels (Figure 7C and Supplementary Figure S11A). When the analysis was performed on a larger region from TSS (±30 kb), the correlations between variations in H3K27ac levels and transcriptional response to NKL54 treatment were even more pronounced (Figure 7D and Supplementary Figure S11B). Analysis of absolute H3K27ac levels shows that genes that are upregulated in response to NKL54 are characterized by lower H3K27ac signals, whereas genes that are downregulated after NKL54 treatment, exhibit higher acetylation levels within a 3kb as well as 30kb around TSS (Figures 7E, F and Supplementary Figure S11C, D).

To confirm this observation, we compared the TPM of genes up and downregulated in response to NKL54. Genes that were not regulated by the inhibitors served as reference. Figure 7G demonstrates that genes upregulated by NKL54 are expressed at low levels, whereas downregulated genes are highly expressed genes. Similar behaviors can be observed for SAHA, for MC2984 after 24 h of treatment and in MC2985, but only after 6 h. TMP195 does not show these differential effects, which further corroborates its distance from the ‘classical’ HDACIs (Supplementary Figure S12).

In summary, NKL54 promotes the transcription of weakly expressed genes and antagonizes the expression of highly expressed genes, and these effects correlate with sculpting of the H3K27ac epigenome around the TSS.

### NKL54 modifies the genomic distribution of MEF2D, HDAC4 and HDAC9

Having shown that NKL54 and other PAOA derivatives can to some extent affect the MEF2-dependent genetic program in LMS, we investigated the influence of NKL54 on the genomic activities of MEF2. We performed MEF2D ChIP-seq using chromatin isolated from SK-UT-1 cells treated with NKL54 for 14 h (Supplementary Figure S10). Under both conditions, a high percentage of MEF2D peaks co-localize with H3K27ac peaks in the presence of NKL54 (Supplementary Figure S13A and B).

**Figure 8.**
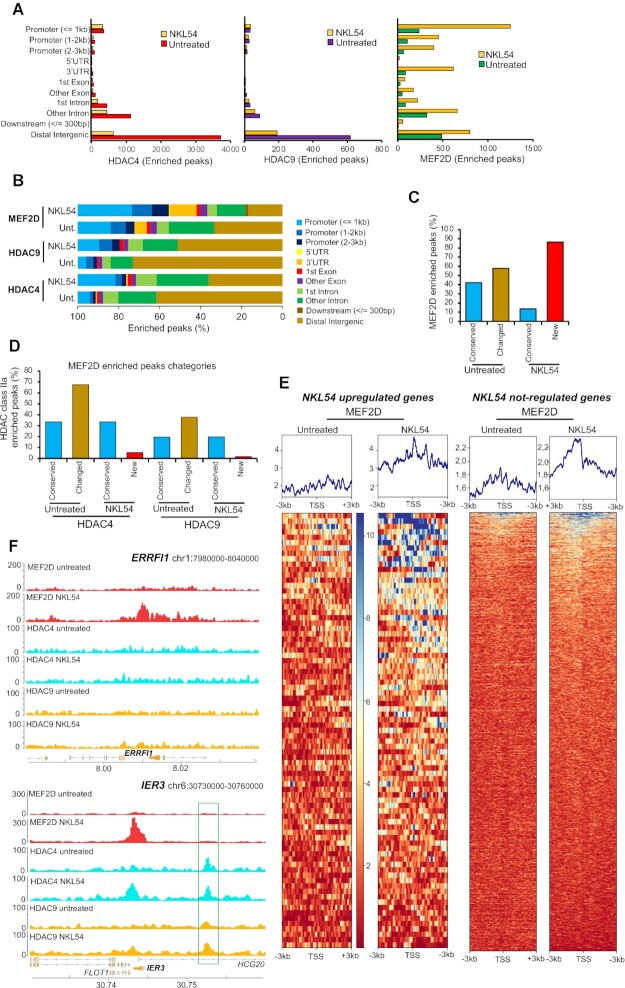
NKL54 exerts a profound influence on the genomic binding of MEF2D, HDAC4 and HDAC9. (**A**) Genomic distribution of HDAC4, HDAC9 and MEF2D-enriched IDR-defined peaks identified by MACS2 in SK-UT-1 cells treated (n = 1770, 393 and 4723) or untreated (*n* = 6109, 891 and 1496) for 14 h with 5 μM NKL54. (**B**) As in panel A, with values represented as percentage. (**C**) Proximity, expressed as percentage of the overall ChIP-seq enriched peaks, between the MEF2D enriched peaks identified in SK-UT-1 cells treated with NKL54 and those found in untreated cells. We defined as ‘Conserved’ the overlapping peaks between cells treated and untreated with NKL54, whereas ‘Changed’ are peaks found only in untreated cells and ‘New’ are peaks found only in NKL54-treated cells. (**D**) Proximity to MEF2D peaks, expressed as percentage, of HDAC4 and HDAC9 ChIP-seq enriched peaks in SK-UT-1 cells treated or untreated with NKL54. MEF2D peaks are defined by the categories showed in panel C. The maximum distance to define overlapping peaks (Conserved) is 1 kb. (**E**) Heat-maps of the MEF2D signal distribution in (left) a region of ±3 kb around the TSS of 90 genes upregulated by NKL54 treatment and showing the appearance of NKL54 *de novo* MEF2D peaks, and (right) around the TSS of 2000 genes not regulated by NKL54 treatment, as indicated. MEF2D signals are compared between untreated and NKL54 treated cells. ChIP-seq data are from experiment 1. (**F**) Detailed view of the MEF2D, HDAC4 and HDAC9 tracks at two representative loci (*ERRFI1* and *IER3*), upregulated by NKL54 and showing *de novo* MEF2D peaks. Gene structure and chromosomal location are shown.

To also evaluate changes in chromatin binding of class IIa HDACs, ChIP-seq experiments were performed for HDAC4 and HDAC9 under the same conditions (Supplementary Figure S10). We observed that HDAC4 and HDAC9 show similar behavior in terms of genome binding. NKL54 causes a reduction in the binding of HDAC4 and HDAC9, especially in the intergenic regions. Instead, their binding to promoters is conserved (Figure 8A and B). In contrast, NKL54 causes a dramatic increase in MEF2D binding to several genomic regions and especially to promoters (Figure 8A and B). These new NKL54-enhanced MEF2D-chromatin interactions are often located at new genomic regions (nearly 90% of the enriched peaks are new) (Figure 8C ‘new’), whereas 40% of MEF2D peaks in untreated cells are conserved in NKL54-treated cells (Figure 8C ‘conserved’). Importantly, in these new regions, MEF2D is frequently recruited in the absence of HDAC4 or of HDAC9 (Figure 8D). In contrast, in 33,4% of MEF2D peaks conserved between untreated and treated cells, HDAC4 is present and HDAC9 is found in approximately 20% of these regions. Therefore, NKL54 promotes the binding of MEF2D to numerous and novel genomic regions and within these regions it should act as a transcriptional activator as HDAC4 and HDAC9 are not recruited.

Interestingly, enriched MEF2D peaks are found in the promoter of 90 genes upregulated by NKL54. To further support this observation, we compared the effect of NKL54 on the genomic binding of MEF2D to the promoters of these 90 genes with respect to the 2000 genes that show no variation in their expression (Figure 8E and Supplementary Figure S13C). MEF2D binding is strongly increased by NKL54 at the promoter regions of the 90 upregulated genes, whereas the effect is much smaller/absent at the promoters of the not-regulated genes. Two different loci (*ERRFI1* and *IER3*) encoding genes regulated by MEF2D and by NKL54 are a good example of these changes. In *ERRFI1*, NKL54 triggers MEF2D to bind to a large genomic region in the absence of HDAC4 and of HDAC9. Instead, at the *IER3* locus, NKL54 promotes binding of MEF2D as well as of HDAC4 and, to a smaller extent, of HDAC9 (Figure 8F and Supplementary Figure S14A). This observation further suggests that NKL54 cannot affect binding between MEF2 and class IIa HDACs *in vivo*. Interestingly, in another region near the locus, binding of both HDAC4 and HDAC9 can be detected, and this binding is independent from MEF2D and NKL54 (Figure 8F and Supplementary Figure S14A, highlighted in green). Two other examples of loci whose expression is upregulated by NKL54 treatment and characterized by the appearance of MEF2D binding after NKL54 treatment are *CXCL1* and *CXCL2* (Supplementary Figure S14B).

## DISCUSSION

The availability of small compounds that induce chromatin remodelling in neoplastic cells is a promising anticancer strategy. The development of small molecules that alter protein-protein interactions is a challenging but also a new and growing area of drug discovery. Here, we have investigated and characterized the possibility of disrupting the interaction between MEF2 and class IIa HDACs. The original idea was to target the surface of the interaction between MEF2 and these epigenetic repressors in LMS. However, because the MEF2-HDAC axis is also perturbed in other cancers, our study may have much broader implications (1,17).

The prototype molecule was the PAOA derivative NKL54 (9). Molecular modelling confirmed that NKL54 should be able to fit into the hydrophobic groove of MEF2. Unfortunately, our *in vitro* and *in vivo* studies show that NKL54 could not compete with the binding between HDAC4 and MEF2A/D, although the binding, at least *in vitro*, is dynamic. Several hypotheses can be formulated. Structural deficiencies in mimicking α-helix distribution, limited contact sites within the hydrophobic groove, or failure to release the hot-spots of protein-protein interactions may explain the inability of NKL54 to act as an orthosteric inhibitor of MEF2-HDACs interactions (49,50). MC2984 and MC2985, predicted by molecular modelling to interact with MEF2 similarly to NKL54, are also unable to compete with HDAC4 peptide binding. Importantly, only compounds that inhibit KDACs and increase histone acetylation can induce LMS cell death.

The persistence of HDAC inhibitory activity in these PAOA derivatives was confirmed by comparative transcriptomic analysis. Although these compounds, unlike SAHA, do not inhibit HDAC6 and HDAC8, the DEGs are largely overlapping with those found for SAHA. Thus, in SK-UT-1 cells, inhibition of HDAC1/HDAC2/HDAC3 causes most transcriptional adjustments and is sufficient to trigger cell death. A group of pro-apoptotic BCL2 members belonging to the BH3-only subfamily are upregulated by SAHA and PAOA derivatives, providing a link between HDAC1/2/3 inhibition and their upregulation. The expression of BIM, BMF and HRK is strongly upregulated as an early response and high levels are maintained throughout. Similarly, BCL2L11/BIM and BBC3/PUMA are upregulated, although less strongly. All these BH3-only members are upregulated by different HDACIs in different cancer models (51–55).

In general, upregulated genes are expressed at low levels in untreated cells, whereas downregulated genes are abundantly expressed. Moreover, the downregulated genes characterize the late response to HDACIs. This repressive wave may represent an adaptation to the unscheduled transcriptional reprogramming. The downregulation of several KATs can also be seen in this context (46,56,57).

The repressive effect of HDACIs on highly transcribed genes may be due to different mechanisms. HDACs may limit acetylation in the gene body and intergenic regions (58,59). This action optimizes recruitment of BRD4, a key elongation factor at promoters and enhancers (59). HDACIs can block elongation of RNA polymerase II and increase pausing of RNAPII at enhancers and super-enhancers (60). At super-enhancers, HDACIs can also cause excessive H3K27ac spreading, an effect that alters normal chromosomal looping (61). Erosion of super-enhancers boundaries, because of H3K27ac spreading, may also be responsible for downregulation of highly expressed genes.

Among PAOA derivatives, NKL54 and MC2984 show few differences. In general, NKL54 is more potent and modulates more genes, especially those that are downregulated. The mechanism through which, the trifluoro group can cause such differences deserves further investigation. Curiously, but expected (39,62), high concentrations of NKL54 can inhibit HDAC4 *in vitro*.

Neural differentiation represents the most enriched DEGs category in response to SAHA and PAOA. Interestingly, gene programs related to neural differentiation have also been activated in other cell lines: synovial sarcoma cells, human embryonic stem cells, and malignant rhabdoid tumor cells, in response to structurally unrelated HDACIs (54,63). They may represent a genetic program silenced by HDACs in non-neuronal cells and reactivated in the presence of the inhibitors.

Only few genes are modulated by MC2985. This compound is a very weak HDAC inhibitor but has strong pro-death activity. Therefore, it is plausible that MC2985 has additional targets, possibly through the action of its 2(alkylthio)-4-phenyl-pirimidine group.

TMP195 is a class IIa specific inhibitor. In LMS cells, it shows a weak anti-proliferative effect. In contrast, deletion of HDAC4 and of HDAC9 strongly affects cell survival and proliferation (19,36). The role of these epigenetic regulators, as scaffolds for the assembly of multiprotein complexes, may explain this discrepancy (64,65). Catalytic domain targeting may not be sufficient to knock down all class IIa activities. Approximately 50% of the genes modulated by TMP195 are also modulated by class I HDAC inhibitors. This overlap is not surprising because class IIa enzymes coordinate the activity of the NCOR1-NCORII-HDAC3 complex via the deacetylase domain (66).

We have shown that HDACIs and NKL54 particularly, can affect MEF2 transcriptional activity. First, HDAC7 (at earlier times) and, HDAC4 and HDAC9 (later) are downregulated. Second, MEF2C and MEF2D are upregulated. These changes could contribute to convert MEF2 complexes dedicated to repression into transcriptional activators. Indeed, approximately 30% of genes under MEF2 regulation are also upregulated by NKL54. Consistent with our observations, BML-210 can promote the activation of MEF2-dependent memory-related genes and the increase of synaptic markers in the hippocampus of a mouse model of Huntington's disease (67).

ChIP-seq experiments have revealed a global increase in MEF2D genome occupancy in response to NKL54. Increased recruitment of TFs to regulatory regions in response to HDACIs has been reported for PU.I (68). In the case of MEF2D, further studies will be necessary to clarify the effect of NKL54 on MEF2D genome occupancy. The creation of new and more accessible chromatin regions could be evoked (69), but a direct effect on MEF2D acetylation status and potentiation of its DNA-binding activity cannot be excluded. Indeed, it has been reported that HDAC3 can bind and deacetylate MEF2D (70–72).

In conclusion, upregulation of the MEF2 transcriptional program may be beneficial for LMS patients, as evidenced by the better prognosis when the MEF2-NKL54 signature is expressed at higher levels. Targeting the interaction between MEF2 and class IIa HDACs is still an open challenge. Our results further stimulate the search for new compounds capable of reactivating MEF2-dependent transcription.

## DATA AVAILABILITY

The transcriptomic raw data are available as GEO accession GSE180804: https://www.ncbi.nlm.nih.gov/geo/query/acc.cgi?acc=GSE180804.

Raw data corresponding to ChIP-seq experiments are uploaded with GEO accession GSE180681: https://www.ncbi.nlm.nih.gov/geo/query/acc.cgi?acc=GSE180681.

The link to a UCSC genome browser session displaying the uploaded sequence tracks is https://genome.ucsc.edu/s/DameBioinfo/NAR_2021_rep.

## Supplementary Material

gkac081_Supplemental_Files
