# Supplementary material for: Transcriptomic and genomic studies classify NKL54 as a histone deacetylase inhibitor with indirect influence on MEF2-dependent transcription
Source: Nucleic Acids Res. 2022 Feb 12;50(5):2566–86. doi: 10.1093/nar/gkac081 (PMC8934631; doi:10.1093/nar/gkac081)
Supplement: gkac081_Supplemental_Files [file gkac081_supplemental_files.zip › SUPPLEMENTARY DATA rev.pdf]

SUPPLEMENTARY DATA

Transcriptomic and genomic studies classify NKL54 as a histone deacetylase inhibitor with indirect influences on MEF2-dependent transcription

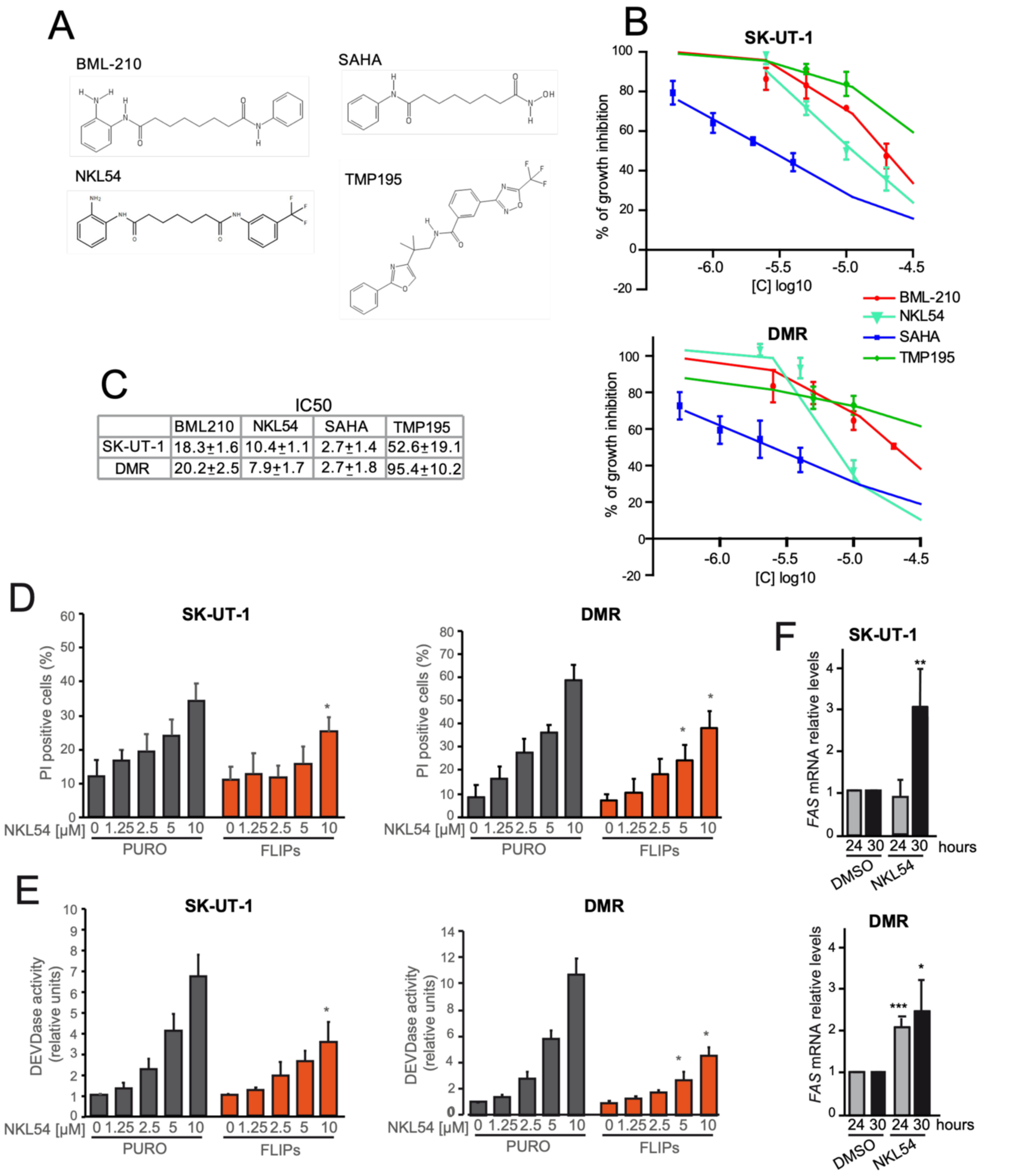

	BML210	NKL54	SAHA	TMP195
SK-UT-1	18.3±1.6	10.4±1.1	2.7±1.4	52.6±19.1
DMR	20.2±2.5	7.9±1.7	2.7±1.8	95.4±10.2

Figure S1. Antiproliferative activities of different molecules targeting the MEF2-HDAC axis in LMS cell lines.

A) Chemical structures of different small molecules that target the MEF2-HDAC axis. The non-selective HDAC inhibitor SAHA was used as control.

B) The resazurin assay was applied to calculate the IC50 of BML210, NKL54, TMP195 and SAHA, as control, in SK-UT-1 and DMR cells. Cells were treated for 40h with serial dilutions

of the compounds: BML210 (2.5µM, 5µM, 10µM, 20µM), NKL54 (2.5µM, 5µM, 10µM, 20µM), TMP195 (5µM, 10µM, 20µM, 40µM) and SAHA (0.5µM, 1µM, 2µM, 4µM).
C) IC50 values for the different inhibitors as calculated from experiments as described in B).
D) Cytofluorimetric analysis of cell death in SK-UT-1 and DMR cells expressing or not FLIPs and treated for 36h with the indicated concentration of NKL54. Data are from 4 independent experiments, + S.D. Asterisks point to the statistical validation between cells expressing or not FLIPs
E) Caspase assay in SK-UT-1 and DMR cells, expressing or not FLIPs and treated as is D). Data are from 3 independent experiments, + S.D. Asterisks point to the statistical validation between cells expressing or not FLIPs.
F) mRNA expression levels of FAS were measured by qRT-PCR after 24h and 30h of the indicated drug treatment in SK-UT-1 and DMR cells. HPRT was used as housekeeping gene.

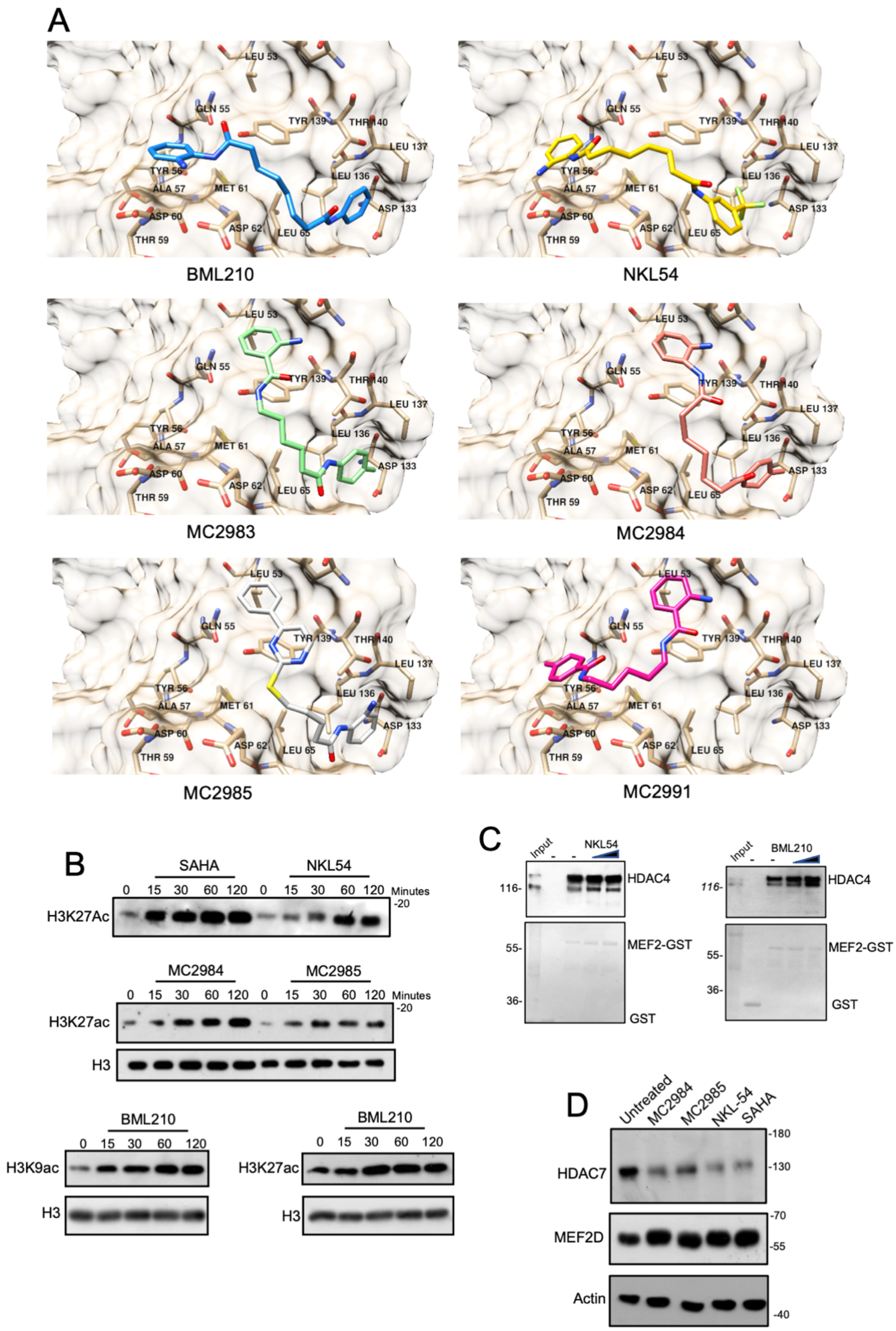

Figure S2. Characterization of small compounds targeting the interaction between MEF2 and class IIa HDACs.

A) BML-210, NKL54, (MC2983, MC2984, MC2985 and MC2991 docked conformations in the MEF2A HDAC4 binding site (PDB entry code 3MU6) hydrophobic groove. Plants/PLP combination as implemented in 3d-qsar.com was used to dock the compounds. Lowest docked

conformation was imported in UCSF Chimera along with the cleaned minimized MEF2A (lock) for binding mode inspection and comparison.
B) Immunoblotting analysis of H3K27 acetylation and total H3 levels in SK-UT-1 cells treated with the indicated compounds for the indicated minutes. Concentrations were SAHA [2,5µM] and [5µM] for all the other compounds
C) GST pull-down assay, using recombinant MEF2D (1-190) or GST as control. Purified GST or GST-MEF2D recombinant proteins (2µg) were incubated with cellular lysates obtained from NIH3T3 cells overexpressing HDAC4 mutated in 14-3-3 binding sites. This mutant cannot be phosphorylated and nuclear exported, thus increasing the pool of HDAC4 available for MEF2D binding. Two different concentrations 14µM and 42µM of NKL54 were used. For BML-210 concentrations were further raised to reach 10x and 100x in respect to GST-MEF2D. Immunoblots were performed to visualize HDAC4. GST and GST-MEF2D were visualized with Ponceau S staining.
D) Immunoblot analysis of MEF2D and HDAC7 levels in SK-UT-1 cells treated with the indicated compounds for 24 hours. SAHA was used at 2.5µM, NKL54 at 5µM, MC2984 and MC2985 at 10µM. Actin was used as loading control.

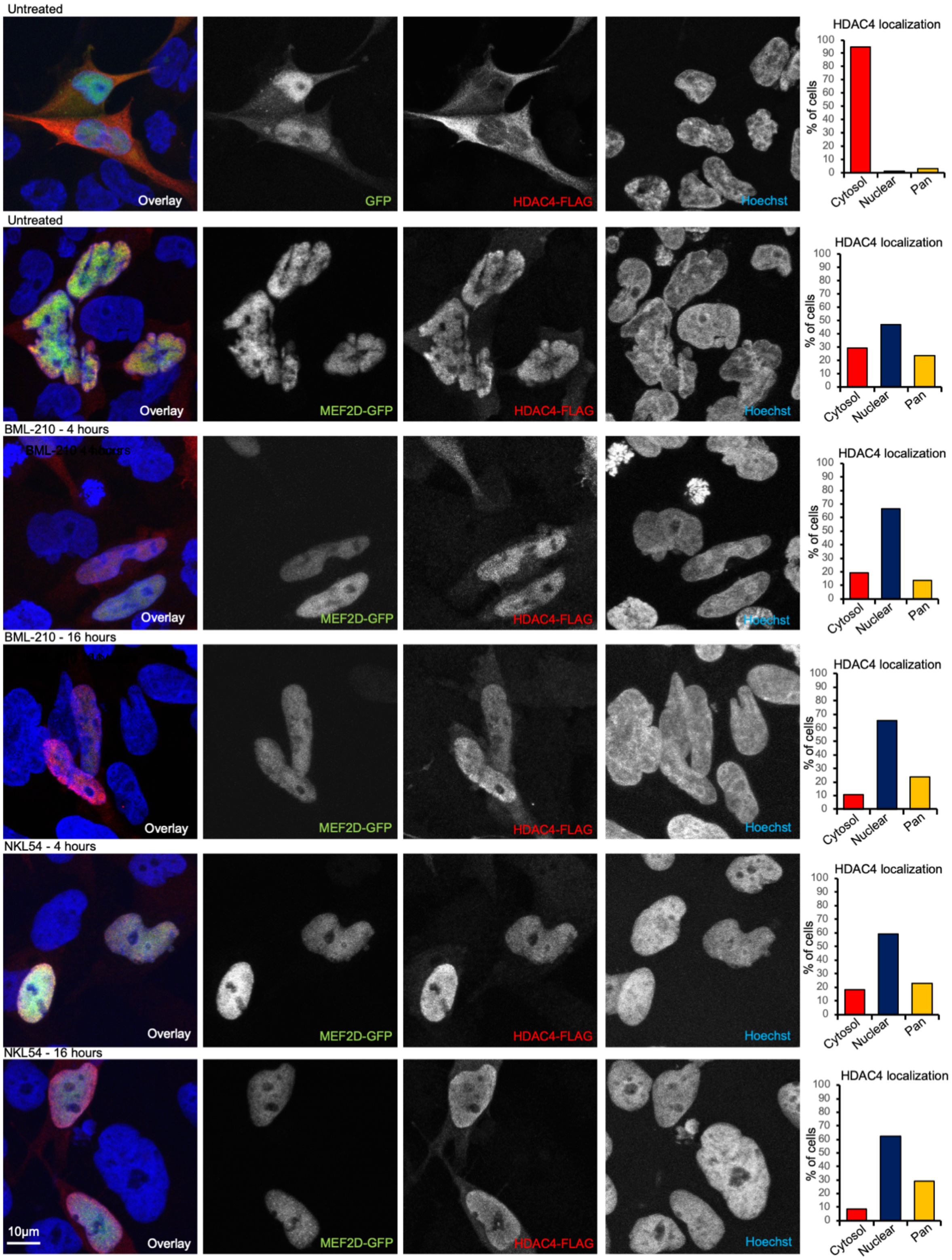

Figure S3. Subcellular localization of MEF2D and HDAC4 in BML-210 and NKL54 treated cells.

MEF2D-GFP and HDAC4-FLAG were ectopically expressed in SK-UT-1 cells. After 12 hours from transfection, cells were treated for 4h and 16h with 5µM of NKL-54 or BML-210 or DMSO as control. Next, cells were fixed with 3% PFA, processed for immunofluorescence and stained with anti-HDAC4 antibodies. As secondary antibody a goat anti-rabbit conjugated

to 633-Alexa-Fluor-conjugated (Life Technologies) was used. Nuclei were visualized with Hoechst 33342. Confocal images were taken using a Leica SP8 LSM and the counting of HDAC4 localization (cytoplasmic, nuclear or pan) is reported by the histograms.

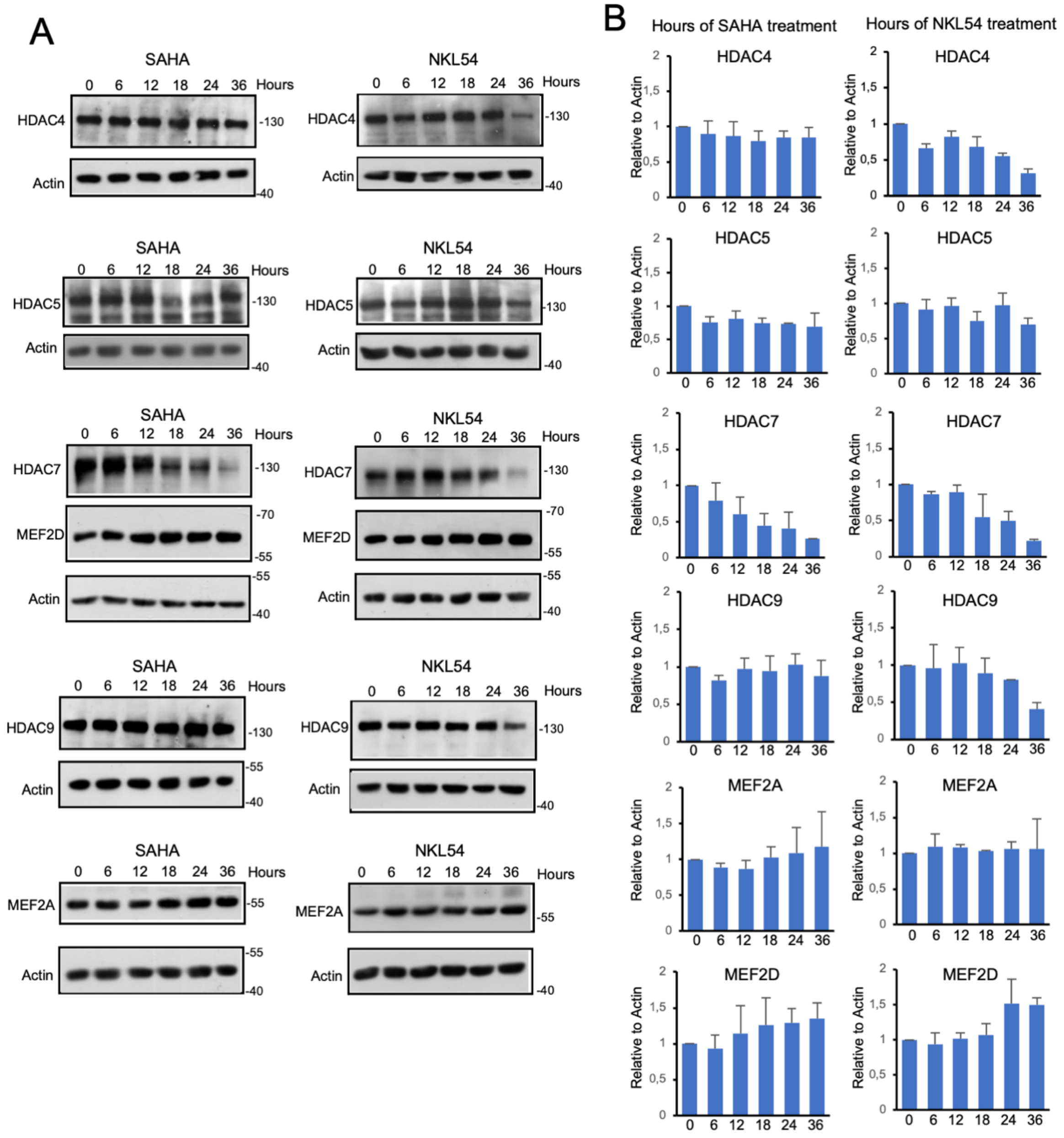

Figure S4. Regulation of MEF2s and class IIa HDACs levels in response to different HDACIs.

A) SK-UT-1 cells were treated for the indicated times with NKL54 [5µM] or SAHA [2,5µM]. Cellular lysates were generated and immunoblot performed using the indicated antibodies. Actin was used as loading control.

B) The densitometric analysis of immunoblots showed in Figure S4A and in Figure 3B is included.

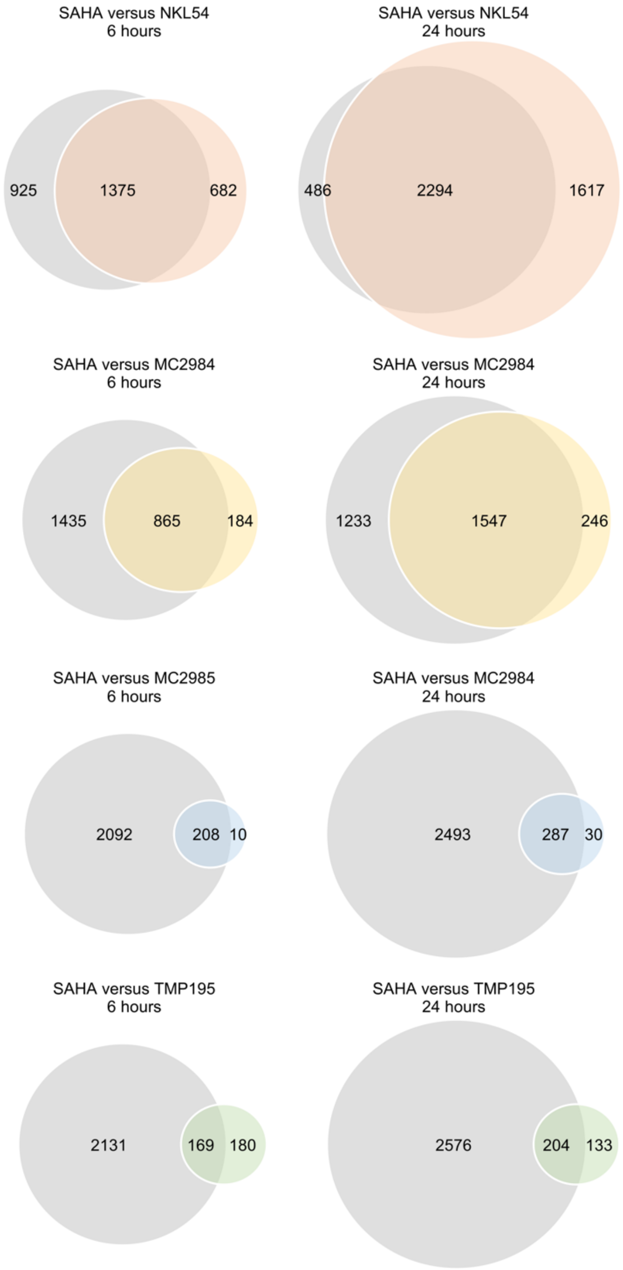

Figure S5. The transcriptional adaptations elicited by the different compounds evidence a major role as HDACIs.

Venn diagrams showing the overlaps between DEG modulated by SAHA and DEG modulated by the different HDACIs at the indicated hours.

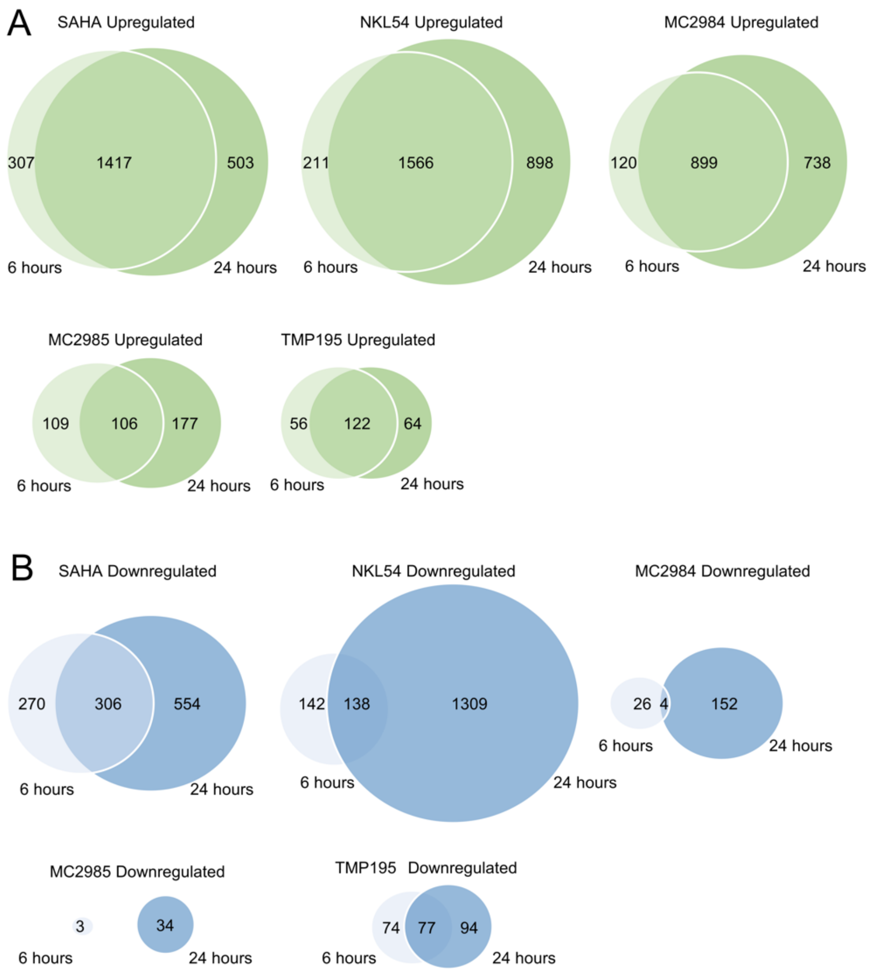

Figure S6. Early, maintained and late transcriptional responses to the different HDACIs.
A) Venn diagrams showing the overlaps between genes upregulated by the different HDACIs after 6 and 24 hours of treatments.
B) Like panel A, instead for downregulated genes.

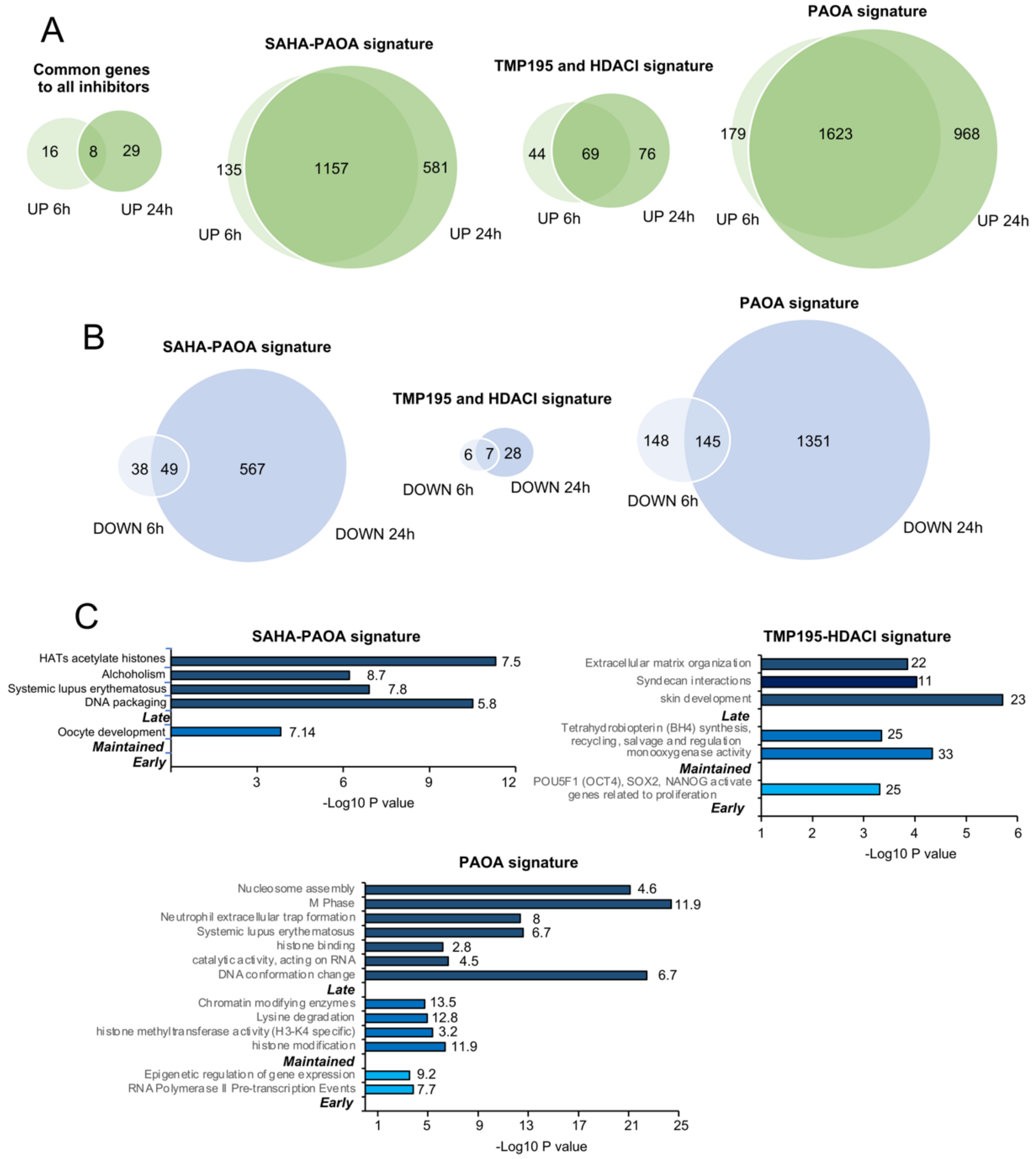

Figure S7. Identification and characterization of genetic signatures marking common and specific responses to the different HDACIs.

A) Venn diagrams showing the overlaps between genes upregulated by the indicated signatures after 6 and 24 hours of treatments.

B) Like panel A, instead for downregulated genes.

C) Bar plots of the most significantly enriched functional terms according to the GO: Biological Process, GO: Molecular Function, Reactome or KEGG databases. The analysis was performed for the indicated downregulated compound-specific gene signatures, retaining the top terms for each functional database. Numbers to the right of the bars represent the percentage of significantly enriched genes found within each category.

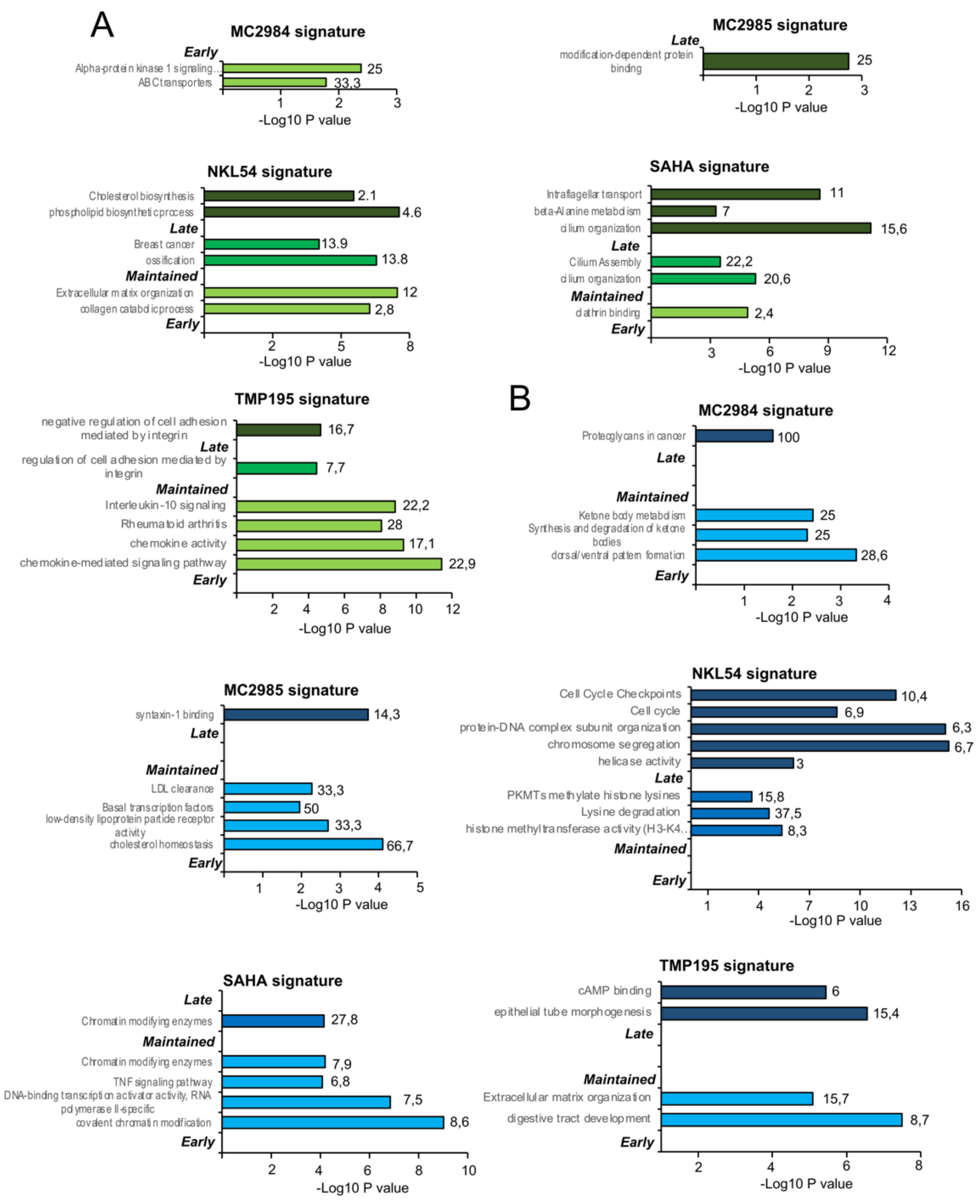

Figure S8. The specific transcriptional modifications induced by the different HDACIs. A) Bar plots of the ClusterProfiler-ReactomePA most significantly enriched functional terms according to the GO: Biological Process, GO: Molecular Function, Reactome or KEGG databases. The analysis was performed for the indicated compound-specific upregulated genes, retaining the top terms for each functional database.

B) Same as panel A, instead for compound-specific downregulated genes.

Numbers to the right of the bars represent the percentage of significantly enriched genes found within each category.

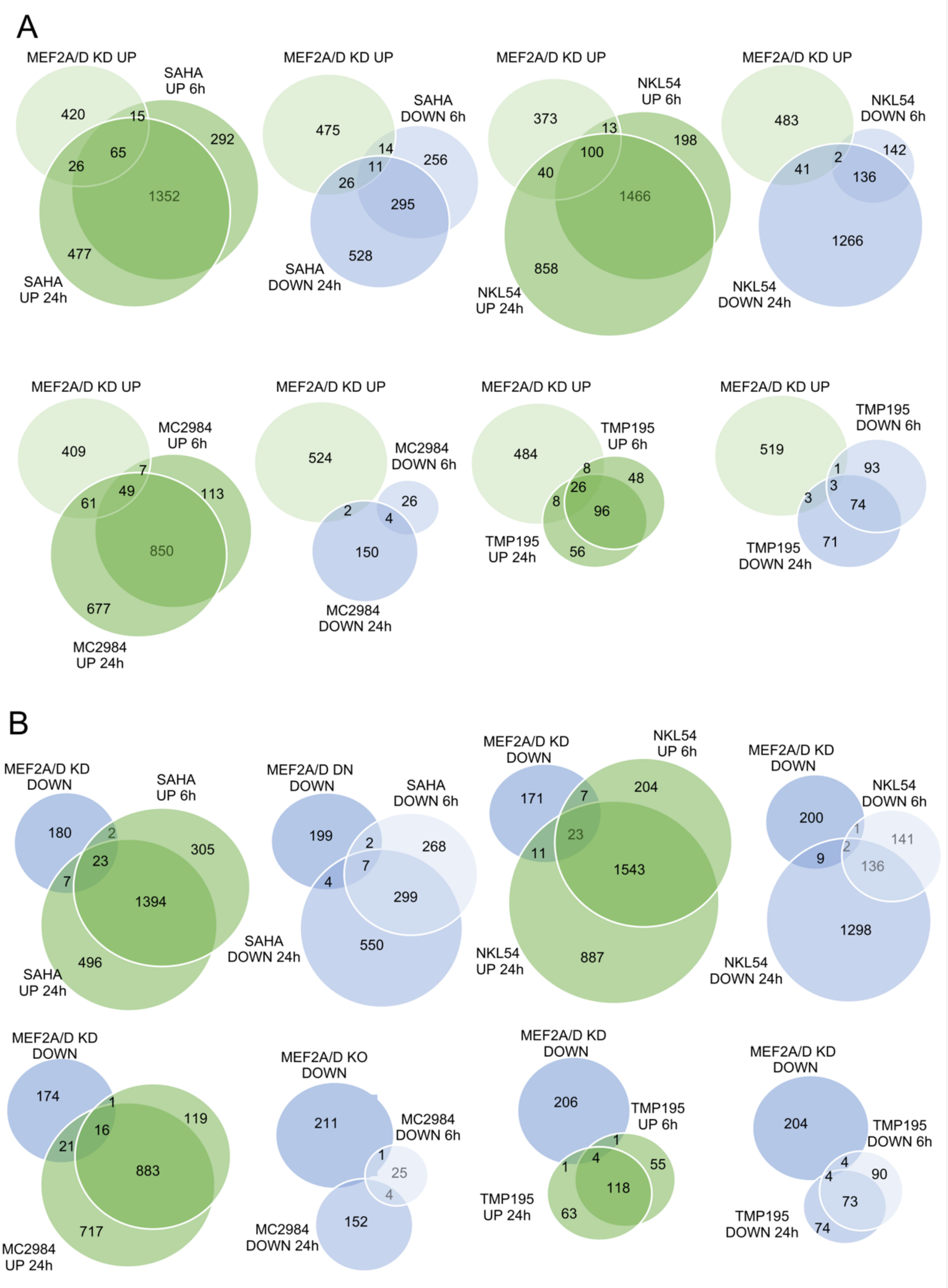

Figure S9. HDACIs influence the expression of genes regulated by MEF2 in LMS cells.
A) Venn diagrams showing the overlaps between genes upregulated after MEF2A or MEF2D silencing in SK-UT-1 cells and genes regulated by the different HDACIs in the same cells at the indicated signatures after 6 and 24 hours of treatments.
B) Like panel A, instead for downregulated genes.

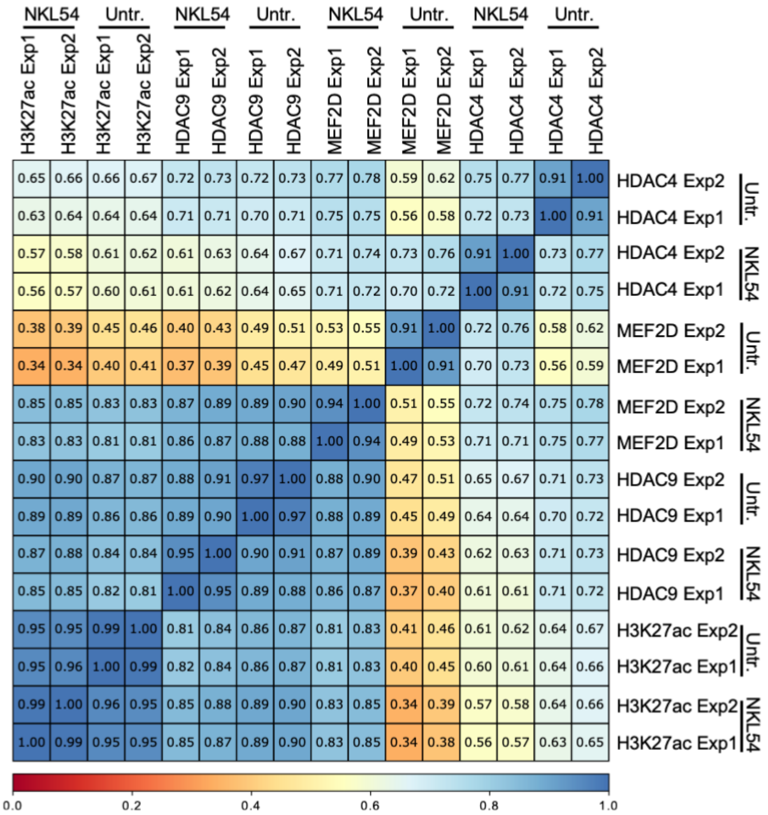

Figure S10. Spearman Correlation between ChIP-seq experiment 1 and experiment 2. ChIP-seq were performed using the indicated antibodies in SK-UT-1 cells treated or not for 14 hours with 5µM NKL54. Average Scores Per Genomic Bin. (10kb). All the replicates always show a very strong correlation (above 0.90).

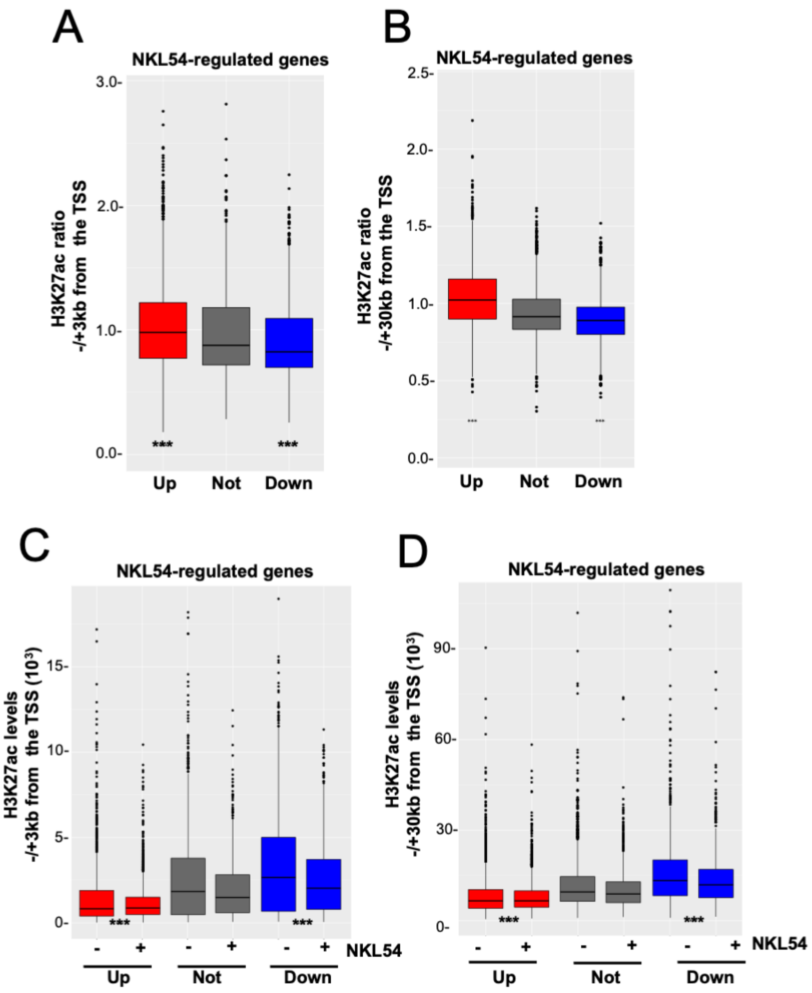

Figure S11. Variation of H3K27ac levels in ChIP-seq experiment 2.

A) H3K27ac ratio between NKL54 treated and untreated cells within ±3kb from TSS. Genes not regulated by NKL54 were selected based on having the lowest combined gene expression variations at 6 and 24 hours from treatment. The boxes indicate the interquartile range with the center line representing the median value. The outliers are plotted as dots. Significance is tested using the Mann-Whitney U test.

B) As in panel A, with H3K27ac ratio between NKL54 treated and untreated cells calculated within ±30kb from TSS.

C) Overall acetylation levels in the ±3kb region centered on the TSS of the indicated gene categories in presence or absence of NKL54. Boxes plotted as in panel A, as well as significance test.

D) As in panel C with the overall acetylation levels measured within a ±30kb region centered on the TSS of the indicated gene categories in presence or not of NKL54.

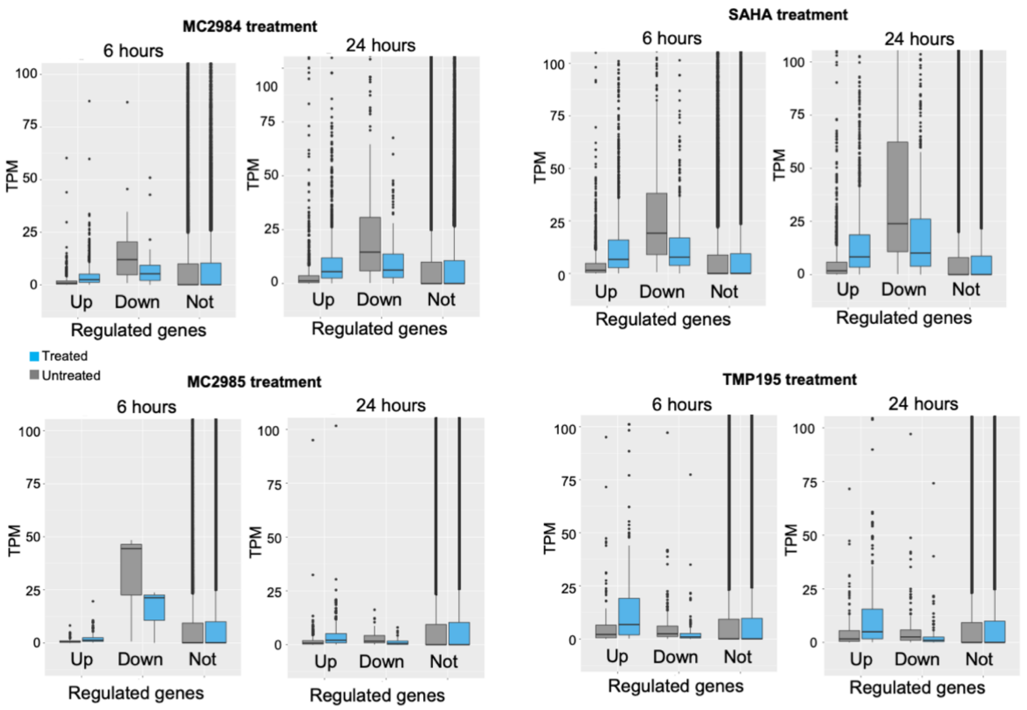

Figure S12. mRNAs abundance of genes regulated by HDACIs.
TPM are shown before and after treatments with the different compounds for the indicated times. TPM were calculated from a gene model where isoforms were collapsed into a single gene.

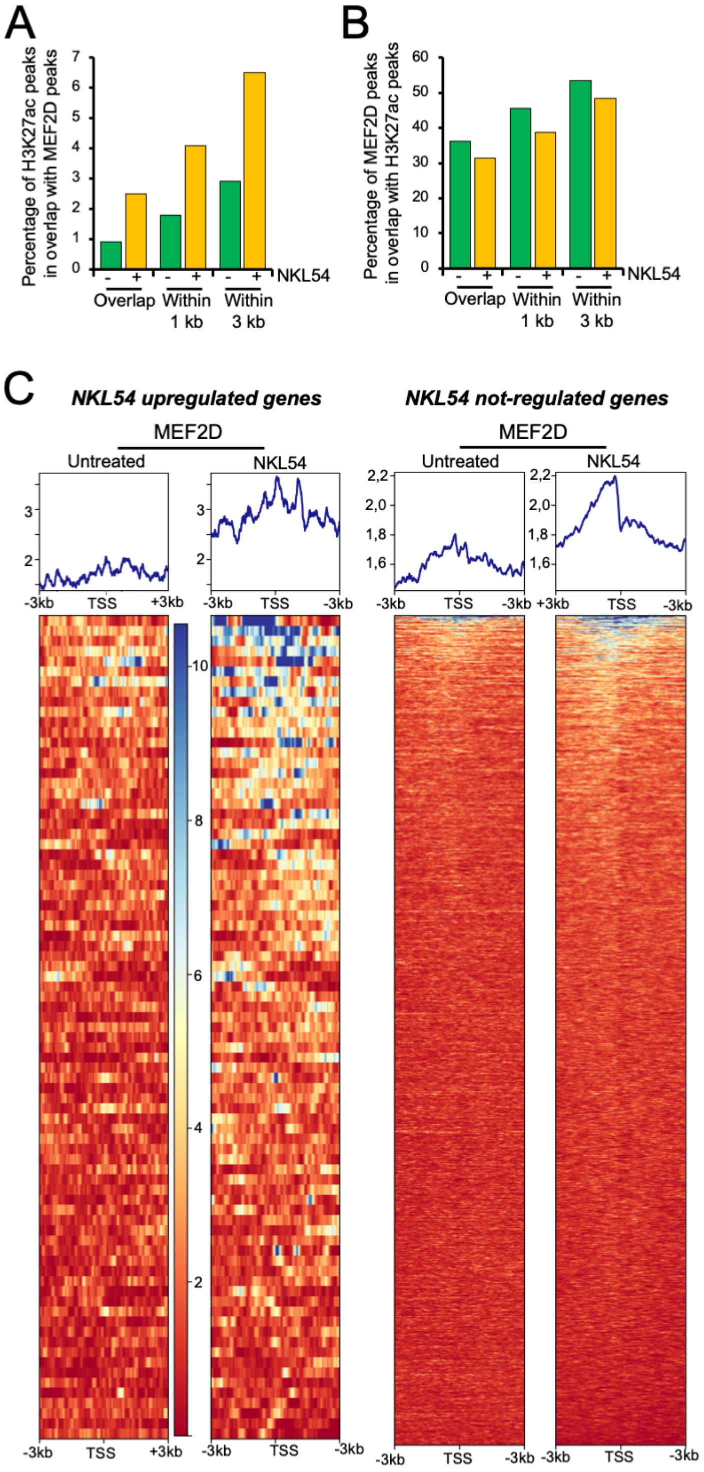

Figure S13. NKL54 exerts a profound influence on the genomic binding of MEF2D, HDAC4 and HDAC9.

A) Percentage of the H3K27ac IDR peaks that overlap MEF2D peaks in cells treated or not with NKL54.

B) Percentage of the MEF2D IDR peaks that overlap MEF2D peaks in cells treated or not with NKL54.

C) Heat-maps of the MEF2D signal distribution in (left) a region of ± 3Kb around the TSS of 90 genes upregulated by NKL54 treatment and showing the appearance of NKL54 de novo MEF2D peaks, and (right) around the TSS of 2000 genes not regulated by NKL54 treatment, as indicated. MEF2D signals are compared between untreated and NKL54 treated cells. ChIP-seq data are from experiment 2.

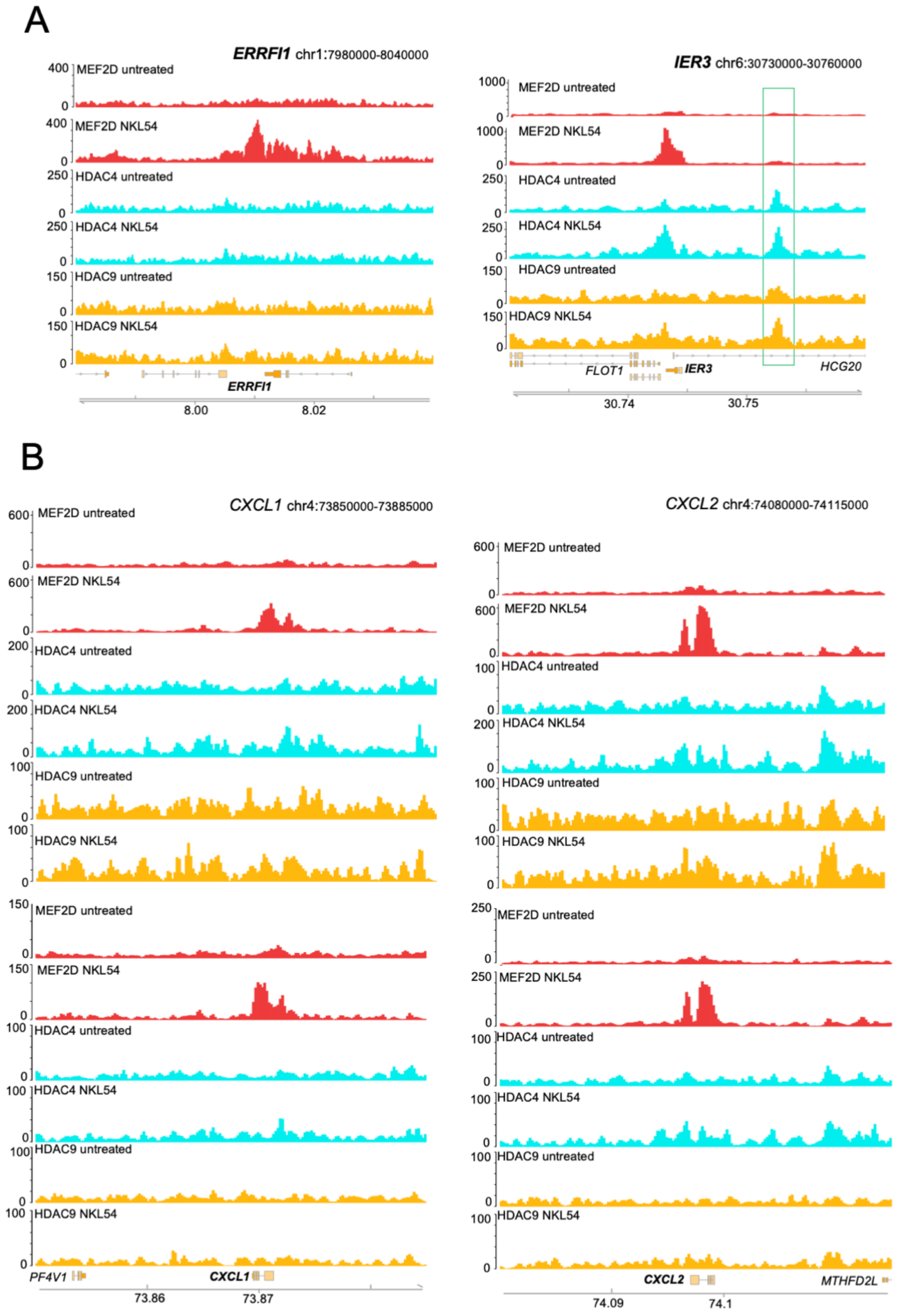

Figure S14. Genomic activities of NKL54 and related compounds

A) Detailed view of the MEF2D, HDAC4 and HDAC9 tracks at *ERRFI1* and *IER3* loci, whose expression is upregulated by NKL54. Data are from ChIP-seq experiment 2. The gene structure and the chromosomal location are shown.

B) Detailed view of the MEF2D, HDAC4 and HDAC9 tracks at *CXCL1* and *CXCL2* loci, whose expression is upregulated by NKL54. In the upper part data are from the ChIP-seq experiment 1. In the lower part data are from experiment 2. The gene structure and the chromosomal location are shown.

Table S1. Docking assessment data. Root mean square deviations (RMSDs) are reported for the MEF2A/DNA/BML-210 complex 3MU6 as obtained with all the program/scoring function combinations.

Docking Program	Scoring Function	RMSDs			
		ECRD[b]		RCRD[c]	
		Docked	Minimized[a]	Docked	Minimized[a]
Smina	VINARDO	7.57	7.57	7.97	7.97
	VINA	5.63	5.63	2.11	2.11
	AD4_SCORING	4.56	4.56	10.17	10.17
Plants	PLP	2.04 (1.97)[d]	NA[e]	2.05 (1.99)[d]	NA
	PLP95	11.16	NA	1.79	NA
	CHEMPLP	2.00	NA	4.77	NA

[a] optimized pose with the Smina minimization feature; [b] Experimental Conformation Re-Docking; [c] Random Conformation Re-Docking; [d] average of three repetitions; [e] Not Available

Table S2: Selectivity profile of NKL54 and SAHA on a panel of HDAC isozymes.

	**IC_{50} (μM)**					
Compound	**HDAC1**	**HDAC2**	**HDAC3**	**HDAC4**	**HDAC6**	**HDAC8**
**NKL54**	0.084 ± 0.004	0.176 ± 0.005	0.047 ± 0.002	35 ± 3	>50	>50
**SAHA**	0.071 ± 0.002	0.164 ± 0.003	0.065 ± 0.002	>50	0.091 ± 0.002	3.9 ± 0.2
